# Constructed Rice Tracers Identify the Major Virulent Transcription Activator-Like Effectors of the Bacterial Leaf Blight Pathogen

**DOI:** 10.1186/s12284-024-00704-0

**Published:** 2024-04-24

**Authors:** Linlin Liu, Ying Li, Qi Wang, Xiameng Xu, Jiali Yan, Yong Wang, Yijie Wang, Syed Mashab Ali Shah, Yongzheng Peng, Zhangfei Zhu, Zhengyin Xu, Gongyou Chen

**Affiliations:** Shanghai Collaborative Innovation Center of Agri-Seeds/State Key Laboratory of Microbial Metabolism, School of Agriculture and Biology, Shanghai Jiao Tong University, Shanghai, 200240 China

**Keywords:** Bacterial leaf blight, EBE-edited tracer, Major TALE, *Xanthomonas oryzae* pv. *oryzae*, Rice

## Abstract

*Xanthomonas oryzae* pv. *oryzae* (*Xoo*) injects major transcription activator-like effectors (TALEs) into plant cells to activate susceptibility (*S*) genes for promoting bacterial leaf blight in rice. Numerous resistance (*R*) genes have been used to construct differential cultivars of rice to identify races of *Xoo*, but the S genes were rarely considered. Different edited lines of rice cv. Kitaake were constructed using CRISPR/Cas9 gene-editing, including single, double and triple edits in the effector-binding elements (EBEs) located in the promoters of rice *S* genes *OsSWEET11a*, *OsSWEET13* and *OsSWEET14*. The near-isogenic lines (NILs) were used as tracers to detect major TALEs (PthXo1, PthXo2, PthXo3 and their variants) in 50 *Xoo* strains. The pathotypes produced on the tracers determined six major TALE types in the 50 *Xoo* strains. The presence of the major TALEs in *Xoo* strains was consistent with the expression of *S* genes in the tracers, and it was also by known genome sequences. The EBE editing had little effect on agronomic traits, which was conducive to balancing yield and resistance. The rice-tracers generated here provide a valuable tool to track major TALEs of *Xoo* in Asia which then shows what rice cultivars are needed to combat *Xoo* in the field.

**Supplementary Information:**

The online version contains supplementary material available at 10.1186/s12284-024-00704-0.

## Introduction

Plants possess a two-tiered innate immune system that includes pattern-triggered (PTI) and effector-triggered immunity (ETI), which contribute to plant defense against pathogens (Minhang Yuan et al. 2021). Some pathogens deliver effectors into plant cells to evade or inhibit PTI and elicit effector-triggered susceptibility (ETS); as a countermeasure, plants utilize resistance (*R*) genes to intercept pathogen effectors and activate ETI. Collectively, the complex interaction between PTI, ETS and ETI has been summarized in a "zig-zag" model (Ngou et al. 2022; Jones and Dangl 2006).

*Xanthomonas oryzae* pv. *oryzae* (*Xoo*), which causes bacterial leaf blight (BB) in rice, can induce the expression of target *S* genes in host plants by secreting transcription activator-like effector (TALE) proteins (Boch and Bonas 2010). TALEs are transcription factors containing repetitive domains that recognize and bind to effector-binding elements (EBEs) that are specific DNA sequences in the promoter regions of *S* genes (Boch et al. 2009; Moscou and Bogdanove 2009; Timilsina et al. 2020). Breeding plant varieties resistant to pathogens is the most effective and economical approach to control disease. To date, 47 genes conferring resistance to *Xoo* have been identified, and 13 cloned *R* genes are associated with TALEs (Jiang et al. 2020; Zhang et al. 2020; Ji et al. 2020; Chen et al. 2021; Huang et al. 2023; Lu et al. 2022). Unfortunately, the constant evolution in pathogen populations weakens the effectiveness of race-specific *R* genes (Li et al. 2020b).

In contrast to *R* genes, only three *S* genes have been identified in rice for bacterial blight susceptibility in the field*.* These susceptibility genes are sugar transport genes. The sugars will eventually be exported transporter (SWEET) genes encode sugar transporters that are needed for both growth and development in plants; however, these genes are often hijacked by pathogens for nutritional needs, which leads to successful infections (Gupta 2020). Five of the 21 *OsSWEET* genes described in rice are known to be induced by major virulence TALEs (major TALEs), and only three *OsSWEET* genes are known to be targeted in the field (Chen et al. 2010; Streubel et al. 2013; Yang and White 2004; Yuan and Wang 2013). Traditionally, differential cultivars of rice containing different R genes have been used to identify races of *Xoo* (Ogawa 1993; Nugroho et al. 2022), but the number of R genes is much higher than the number of S genes. Therefore, S genes are more suitable for constructing near-isogenic lines (NILs) to monitor the variations of *Xoo*, which is critical for successful breeding and deployment of resistant varieties.

Ten major TALEs have been identified in *Xoo* and are known to target EBEs in the promoters of three *OsSWEET* genes. PthXo1 and AvrXa27A are major TALEs that induce the expression of *OsSWEET11a* (*Os8N3/Xa13*) (B. Yang et al. 2006; Xu et al. 2023), whereas PthXo2 and PthXo2-like TALEs (PthXo2A/B/C, PthXo2.1/PthXo2.2, PthXo2_K74_, PthXo2_PXO61_ and Tal5_LN18_) induce the expression of *OsSWEET13* (*Os12N3/Xa25*) (Zhou et al. 2015; Xu et al. 2019). PthXo3, AvrXa7, TalC and TalF are known to induce the expression of *OsSWEET14* (*Os11N3*), (Oliva et al. 2019; Tran et al. 2018; Antony et al. 2010). Furthermore, TalC and TalF only exist in African strains. DNA polymorphisms in EBEs can prevent TALE binding to target promoters. An analysis of 4,726 rice varieties revealed an A/G variation in the EBE recognized by PthXo1, which had a polymorphism frequency of 0.2% and an adenosine insertion in the PthXo3/AvrXa7 EBE occurred with a frequency of 7.7% (Eom et al. 2019). There were ten variations in the PthXo2 EBE with frequencies ranging from 0.03 to 51.16% and these variations complicate the identification of PthXo2-like TALEs (Xu et al. 2019).

Although the intricacies of rice/*Xoo* interactions have been deciphered in many studies (Oliva et al. 2019; Xu et al. 2022, 2019), it remains inconvenient and inefficient to detect major TALEs in *Xoo*. Southern blot hybridization can determine the number and size of putative TALE genes (Khojasteh et al. 2020) but is inaccurate because the comparison is based on size and is not precise. Whole genome sequencing can predict putative TALEs in *Xoo*, however, the quality of the match between two repeat variable di-residues (RVDs) and the EBE nucleotide is confusing and the final pathotype determination still depended on plant materials. NILs based on rice *R* genes have been used to classify races of *Xoo*, but the large number of *R* genes makes it difficult to establish a complete set of materials (Mondal et al. 2014). Translational fusions between *OsSWEET* gene promoters and β-glucuronidase were used to monitor SWEET protein accumulation (Eom et al. 2019), but the identification procedure was complicated, and these materials cannot be used for breeding. The *OsSWEET* genes knockout rice lines were used to determine which *OsSWEET* genes were targeted by specific *Xoo* strains (Eom et al. 2019), however it ignored the role of *OsSWEET* genes in plant growth and development. A simple, rapid method for monitoring *Xoo* and major TALEs remains elusive and is urgently needed.

To identify major TALEs of *Xoo* in Asia, we used CRISPR–Cas9 genome editing to develop a set of rice lines with single, double and triple mutations in the EBEs of *OsSWEET11a*, *OsSWEET13* and *OsSWEET14*. These mutant rice lines can be used to determine *Xoo* major TALEs by phenotyping after inoculation, thus providing a rapid and strategic screen for resistant varieties that can be utilized in the field.

## Results

### Generation of EBE-Edited Rice Lines

**Fig. 1:**
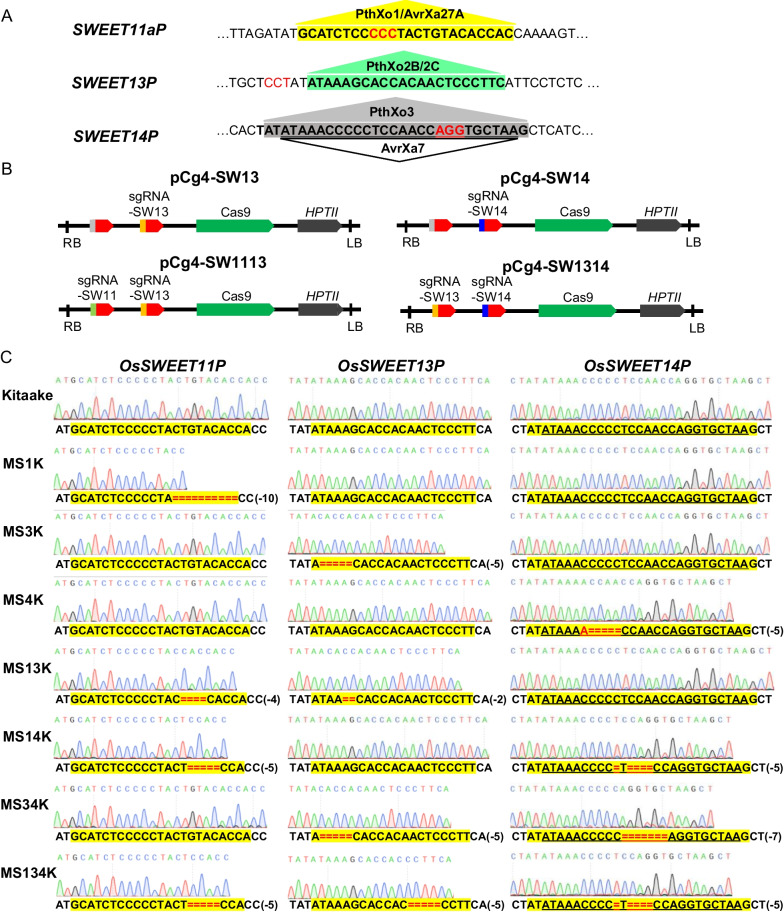
Edited *OsSWEET* EBEs in rice. **A** Schematics of edited EBE sequences recognized by the major virulence TALEs PthXo1/AvrXa27A, PthXo2B/C, PthXo3 and AvrXa7. The PAM region (NGG) is shown in red font. **B** Schematic structures showing Cas9 and guide RNAs for EBE editing in seven mutant lines of cv. Kitaake. The sgRNA target sites are shown in different colors. **C** Nucleotide sequences of *OsSWEET11, OsSWEET13* and *OsSWEET14* promoters and EBEs in cv. Kitaake and seven edited lines. The EBEs recognized by PthXo1, PthXo2B/C and PthXo3 are highlighted in yellow, and the AvrXa7 EBE is underscored

In order to detect major TALEs in virulent *Xoo* strains that cause disease in rice, we generated single, double and triple mutations in the EBEs of *OsSWEET11a*, *OsSWEET13* and *OsSWEET14* using CRISPR–Cas9 technology (Fig. 1A and B). When considered along with wild-type Kitaake rice and mutants previously obtained by our lab (MS14K, Xu et al. 2019; MS1K, Xu et al. 2023), the mutants constitute a set of differential varieties. Regardless of whether the edited site was homozygous, heterozygous or wild-type, it was easily identified by PCR and Sanger sequencing. Homozygous mutants were obtained in the T0 to T3 generations, and mutant lines containing 2–10 nucleotide deletions in EBEs were selected for further study (Additional file 1: Table S1 and Fig. 1C). Mutants with a single edited EBE included MS1K, MS3K and MS4K. MS1K contained a 10-bp deletion in the *OsSWEET11a* EBE, MS3K contained a 5-bp deletion in the *OsSWEET13* EBE, and MS4K contained a 5-bp deletion and an extra adenine nucleotide in the overlapping loci of target EBEs for PthXo3 and AvrXa27 in the *OsSWEET14* promoter (Fig. 1C). Rice lines containing two edited EBEs included mutants MS13K, MS14K and MS34K, whereas MS134K contained mutations in all three EBEs recognized by PthXo1/ PthXo1* (variants of PthXo1, including AvrXa27), PthXo2B/C (here represent PthXo2-like TALEs that are compatible with Kitaake) and PthXo3/PthXo3* (variants of PthXo3, including AvrXa7). For MS134K in this study, we chose a different line with from that reported in Xu et al. 2019 (MS134K-18 and MS134K-19). The line used in the current study contained the appropriate number of base deletions (−5) in *OsSWEET13* EBE (Additional file 1: Table S1). These rice lines constitute a potential series of differential varieties for identification of *Xoo* strains harboring different major TALEs. We speculated that infection of the seven EBE-mutant lines by *Xoo* strains would activate a subset of the *OsSWEET* genes and lead to resistant or susceptible phenotypes, thus identifying what major TALEs were present in a given *Xoo* strain.

### Pathotypes of *Xoo* Strains on EBE-Edited Rice Lines

**Fig. 2:**
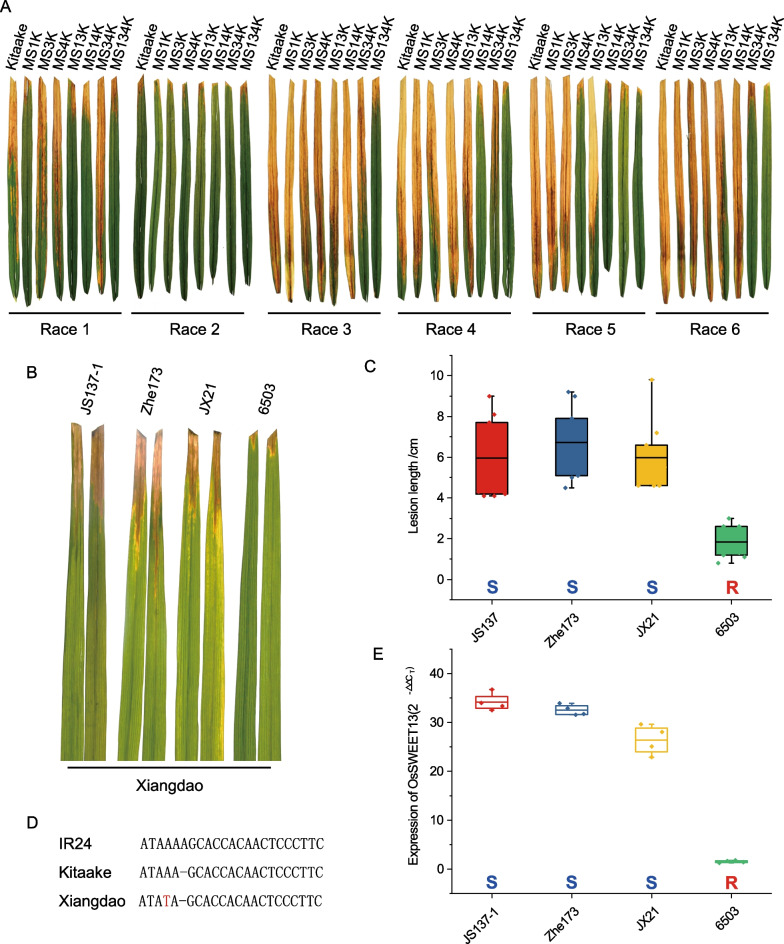
Identification of *Xoo* strains containing only PthXo2B*. **A** Inoculation phenotypes of *Xoo* strains on cv. Kitaake and the edited lines. **B** Disease symptoms on rice cv. Xiangdao inoculated with *Xoo* JS137-1, Zhe173, JX21, and 6503. **C** Mean lesion lengths (cm) induced by *Xoo* JS137-1, Zhe173, JX21, and 6503 on Xiangdao rice (*n* = 10). **D** Polymorphism of *OsSWEET13* EBEs in rice cultivars IR24, Kitaake, and Xiangdao. Hyphens indicate that a single nucleotide was missing in the EBE sequence. **E,** Expression of *OsSWEET13* in rice cv. Xiangdao inoculated with *Xoo* JS137-1, Zhe173, JX21, and 6503 (*n* = 4)

To estimate major TALE prevalence and variation in geographically diverse *Xoo* strains, a collection of 50 *Xoo* strains of Asian (Additional file 1: Table S2) was screened for virulence on cv. Kitaake and its NILs. A tip-cutting method (Ji et al. 2016) was used to identify resistant and susceptible phenotypes in rice lines inoculated with *Xoo* (Fig. 2A, Additional file 1: Table S3 and Table 1). Four of the 50 *Xoo* strains (*Xoo* JS137-1, Zhe173, JX21, 6503) were not virulent on cv. Kitaake or the EBE-edited lines. We speculate that these four strains may contain PthXo2B*, variant of PthXo2B/C that is incompatible with cv. Kitaake, including PthXo2 (Oliva et al. 2019; Xu et al. 2019). In the remaining 46 *Xoo* strains, PXO99^A^ was avirulent on MS1K, which was defective in the *OsSWEET11a* EBE targeted by PthXo1/PthXo1* (or PthXo1/PthXo1* + PthXo2B*). Twenty-eight strains were avirulent on MS4K, which is defective in the *OsSWEET14* EBEs recognized by PthXo3/PthXo3* (or PthXo3/PthXo3* + PthXo2B*). *Xoo* IXO221 was virulent on the single EBE-edited lines MS1K, MS3K and MS4K, but was avirulent on MS14K, which suggests that IXO221 contains PthXo1/PthXo1* and PthXo3/PthXo3* (or PthXo1/PthXo1* + PthXo3/PthXo3* + PthXo2B*). Twelve strains were virulent on single EBE-edited lines, but were avirulent on MS34K, suggesting that these strains harbor both PthXo2B/C and PthXo3/PthXo3*. Four strains (*Xoo* 7914, LN18, LN2, LN4) were virulent on single and double EBE-edited lines and were avirulent on MS134K, which harbors mutant alleles of *OsSWEET11a*, *OsSWEET13* and *OsSWEET14*; this result indicates that these four strains harbor PthXo1/PthXo1*, PthXo2B/C and PthXo3/PthXo3*. The four *Xoo* strains that were unable to cause disease on cv. Kitaake (JS137-1, Zhe173, JX21, 6503) were further studied on the spontaneous mutant lines of OsSWEET13. We speculated that these strains may harbor PthXo2B*. We inoculated five other rice varieties (IR4, ZS97, Minghui63, American Huangkedao, and Xiangdao; Additional file 1: Table S4) with these four strains using the tip-cutting method. *Xoo* JS137-1, Zhe173 and JX21 induced longer lesions on cv. Xiangdao (S) as compared to cv. Kitaake (Additional file 1: Table S5, Fig. 2B and C), and expression analysis indicated that *OsSWEET13* was induced in Xiangdao after inoculation with these three strains (Fig. 2E). When compared to cv. Kitaake, Xiangdao exhibits a single base change in the EBE (Fig. 2D), which confers susceptibility to *Xoo* JS137-1, Zhe173 and JX21 but still resistance to *Xoo* 6503 (Additional file 1: Table S5). An *OsSWEET13* allele compatible with *Xoo* 6503 was not observed in this study, possibly because we have not collected enough alleles or because *Xoo* 6503 is a weakly-virulent strain that is only susceptible under certain environmental conditions.

**Table 1:** Pathotypes of *X*. *oryzae* pv. *oryzae* strains on EBE-edited lines

Strains^c^	Kitaake^a^	MS1K	MS3K	MS4K	MS13K	MS14K	MS34K	MS134K	Race	Major TALEs^b^ predicted by genome sequence	Major TALEs speculated by pathotypes
							
**PXO99**^**A**^	S	R	S	S	R	R	S	R	1	PthXo1	PthXo1
**IXO221**	S	S	S	S	S	R	S	R	2	PthXo1* + PthXo3*	PthXo1/PthXo1* + PthXo3/PthXo3*
6503	R	R	R	R	R	R	R	R	3	ND	PthXo2B*
**JS137-1**	R	R	R	R	R	R	R	R		PthXo2B*	
JX21	R	R	R	R	R	R	R	R		ND	
Zhe173	R	R	R	R	R	R	R	R		ND	
7914	S	S	S	S	S	S	S	R	4	ND	PthXo1/PthXo1* + PthXo2B/C + PthXo3/PthXo3*
**LN18**	S	S	S	S	S	S	S	R		PthXo1* + PthXo2C + AvrXa7	
LN2	S	S	S	S	S	S	S	R		ND	
LN4	S	S	S	S	S	S	S	R		PthXo1* + PthXo2C + AvrXa7	
Oct-78	S	S	S	R	S	R	R	R	5	ND	PthXo3/PthXo3*
AH10	S	S	S	R	S	R	R	R		ND	
GD1052	S	S	S	R	S	R	R	R		ND	
GD9186	S	S	S	R	S	R	R	R		ND	
GWRXoo3	S	S	S	R	S	R	R	R		ND	
GX4	S	S	S	R	S	R	R	R		ND	
GX4	S	S	S	R	S	R	R	R		ND	
HB03-01	S	S	S	R	S	R	R	R		ND	
HB87-7	S	S	S	R	S	R	R	R		ND	
HNZT3	S	S	S	R	S	R	R	R		ND	
HuN37	S	S	S	R	S	R	R	R		PthXo2 + PthXo3*	
KS-1–21	S	S	S	R	S	R	R	R		ND	
LYG48	S	S	S	R	S	R	R	R		ND	
LYG50	S	S	S	R	S	R	R	R		ND	
OS26	S	S	S	R	S	R	R	R		ND	
**PXO86**	S	S	S	R	S	R	R	R		AvrXa7	
SC-4	S	S	S	R	S	R	R	R		ND	
YN04-1	S	S	S	R	S	R	R	R		ND	
YNYM-5	S	S	S	R	S	R	R	R		ND	
IXO191	S	S	S	R	S	R	R	R		ND	
AH28	S	S	S	R	S	R	R	R		PthXo2 + PthXo3	
HNNX3	S	S	S	R	S	R	R	R		ND	
XZ42	S	S	S	R	S	R	R	R		ND	
XZ44	S	S	S	R	S	R	R	R		ND	
YC12	S	S	S	R	S	R	R	R		ND	
JXOV	S	S	S	R	S	R	R	R		ND	
OS86	S	S	S	R	S	R	R	R		ND	
YC1	S	S	S	R	S	R	R	R		ND	
DB11	S	S	S	S	S	S	R	R	6	ND	PthXo2B/C + PthXo3/PthXo3*
DB16	S	S	S	S	S	S	R	R		ND	
DB23	S	S	S	S	S	S	R	R		ND	
DB5	S	S	S	S	S	S	R	R		ND	
DD	S	S	S	S	S	S	R	R		ND	
JL1	S	S	S	S	S	S	R	R		ND	
JL15	S	S	S	S	S	S	R	R		ND	
JL4	S	S	S	S	S	S	R	R		ND	
TH18	S	S	S	S	S	S	R	R		ND	
TH8	S	S	S	S	S	S	R	R		ND	
**PXO61**	S	S	S	S	S	S	R	R		PthXo2B + PthXo3	
GD267	S	S	S	S	S	S	R	R		ND	

^a^Resistant (R) and susceptible (S) phenotypes on rice leaves were determined by lesion lengths on cv. Kitaake and the seven edited lines 14 d after inoculation with *Xoo* strains. ‘S’, denotes mean lesion length > 3.0 cm; ‘R’, indicates mean lesion lengths ≤ 3.0 cm. *Xoo* PXO61, PXO86, PXO99^A^, IXO221, LN4, LN18, AH28 and JS137-1 have been sequenced, and the major virulent TALEs were predicted in the genome sequences. Putative major TALEs were identified based on R and S phenotypes on Kitaake and the edited rice lines. The different shapes and colors of rice lines indicate the different editing sites as follows: squares represent *OsSWEET11a*, circles indicate *OsSWEET13* and pentagons represent *OsSWEET14*. Shapes colored green indicate the presence of wild-type EBEs, and red shapes represent edited EBEs

^b^PthXo1* is a variant of PthXo1, which can activate *OsSWEET11a* expression and cause rice susceptibility. PthXo2* is similar to PthXo2, which can activate the expression of different EBEs in *OsSWEET13* and cause rice susceptibility. AvrXa7 is similar to PthXo3, which activates *OsSWEET14* expression leading to susceptibility. ND, indicates that the major TALEs are unknown (‘not determined’)

^c^The strains in bold font were used as the representative strains of each Race for subsequent *OsSWEET* genes induction expression experiment

### Estimating TALE Diversity Via Genome Analysis

**Fig. 3:**
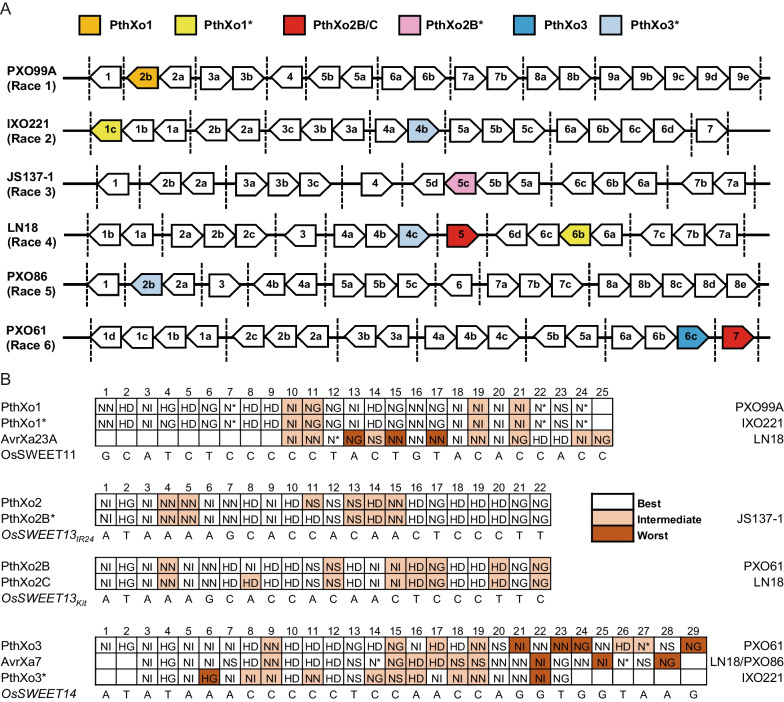
TALE diversity in *Xoo* strains. **A** Predicted major virulence TALEs in the genomes of representative *Xoo* strains from six races. AnnoTALE software was used for TALE prediction. The genomic sequences of PXO99A (race 1), IXO221 (race 2), LN18 (race 4), PXO86 (race 5) and PXO61 (race 6) were obtained from NCBI, and the genomic sequence of JS137-1 (race 3) was generated in our laboratory (unpublished data). **B** Alignment of PthXo1, PthXo3 and PthXo2 in TALE RVD sequences (no gaps allowed) with the DNAsequence of EBEs in the *OsSWEET11, OsSWEET13 and OsSWEET14* promoters. Background colors indicate the quality of the match between the RVDs and the EBE nucleotides (http://bioinfo-web.mpl.ird.fr/cgi-bin2/talvez/talvez.cgi)

Based on the presence of putative major TALEs, the 50 *Xoo* strains were grouped into six races (Table 1). Of these 50 strains, nine had known genome sequences, fortunately, there were strains with known genomes in each group, and the major TALEs predicted by genome sequencing corresponded roughly to phenotypes (Additional file 1: Table S6 and Fig. 3). For example, *Xoo* PXO99^A^ in race 1 encodes only PthXo1, *Xoo* PXO86 in race 5 encodes only PthXo3* (AvrXa7); however, *Xoo* AH28 (race 5) presumably harbors both PthXo2 and PthXo3 based on the genome sequences, but the inoculation phenotype elicited by PthXo2 is not expressed in cv. Kitaake or the NILs. *Xoo* IXO221 in race 2 contains PthXo1* and PthXo3* (Fig. 3). Interestingly, PthXo1* and PthXo3* have not been reported in IXO221, and PthXo1* differs from PthXo1 by only 1 RVD and PthXo3* differs from PthXo3 by 8 RVDs. The predicted target of PthXo3* is different from the EBEs of *OsSWEET14*, so the final phenotype cannot be determined by genomic prediction alone. We then conducted Southern blot analysis on the 50 strains, since this approach has been used to classify fragment of *pthXo1* from *Xoo* PXO99^A^ (Ji et al. 2014). Strains *Xoo* PH (*pthXo1*), PH (*pthXo2*) and PH (*avrXa7*) containing *pthXo1*, *pthXo2* or *avrXa7*, respectively, but lacking other TALEs were used as controls to identify bands encoding *pthXo1*, *pthXo2* or *avrXa7* (Additional file 1: Figure S1). Southern blotting indicated that 47 *Xoo* strains contained putative fragments of *pthXo1*, 46 contained possible fragments of *pthXo2*, 22 contained putative fragments of *pthXo3*, and 23 strains contained fragments of *avrXa7*. Although both PthXo3 and AvrXa7 activated *OsSWEET14*, these two TALEs were rarely present in the same strain. In total, 18 *Xoo* strains contained putative fragments of *pthXo1*, *pthXo2* and *pthXo3*, and 19 strains contained potential fragments of *pthXo1*, *pthXo2* and *avrXa7*, which suggests these strains have the capability to activate *OsSWEET11a*, *OsSWEET13* and *OsSWEET14* simultaneously. It is important to mention that Southern blot results did not correspond to the inoculation phenotypes, possibly because the presence of hybridizing bands in major TALEs indicated the presence of a TALE of similar size that remained unidentified.

### Induction of *OsSWEET* Genes in Rice

**Fig. 4:**
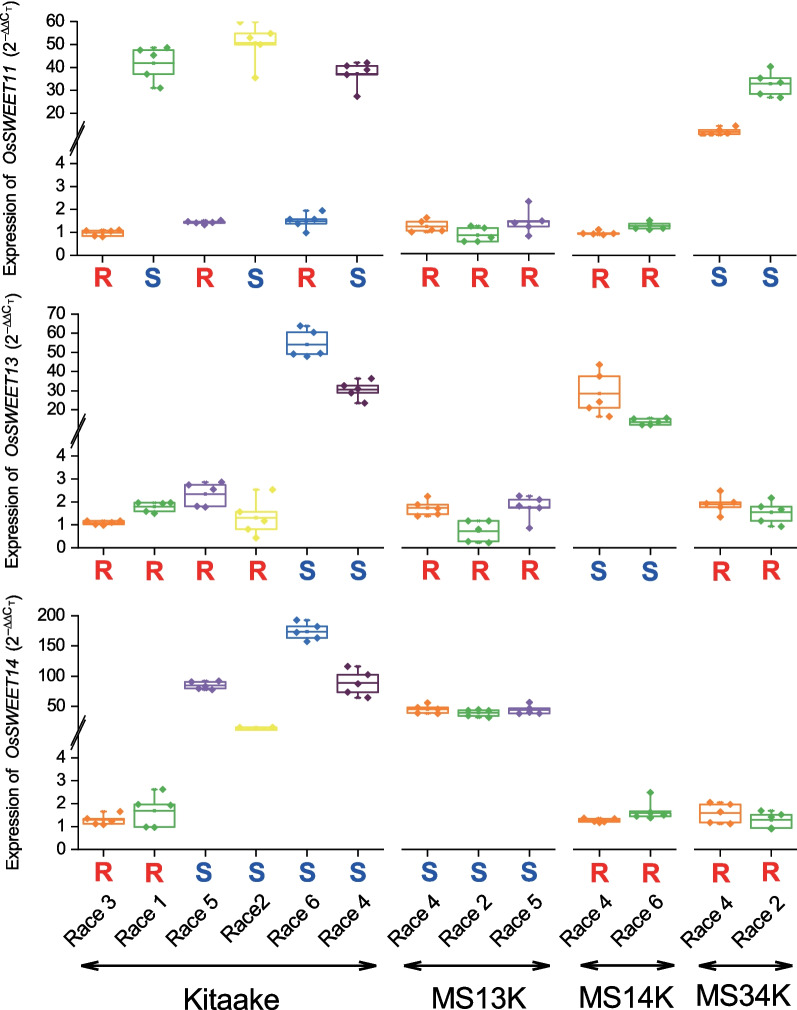
*OsSWEET11*, *OsSWEET13* and *OsSWEET14* induction triggered by PthXo1 (PthXo1*), PthXo2B/C and PthXo3 (PthXo3*) TALEs. qRT–PCR was conducted in rice cv. Kitaake and the edited lines MS13K, MS14K, and MS34K inoculated with *Xoo* PXO99^A^ (race 1), IXO221 (race 2), JS137-1 (race 3), LN18 (race 4), PXO86 (race 5) and PXO61 (race 6). Means and SDs of gene expression relative to a water control were computed from five biological replicates (*n* = 5). Values with the same lowercase letters did not differ significantly at *P* < 0.05 based on ANOVA

To investigate the accuracy of predicted major TALEs, the junctions between diseased and healthy leaves infected with six *Xoo* strains were sampled and *OsSWEET* gene expression was measured (Fig. 4). These six strains were *Xoo* PXO99^A^ (race 1), IXO221 (race 2), JS137-1 (race 3), LN18 (race 4), PXO86 (race 5) and PXO61 (race 6). *Xoo* PXO99^A^ was the sole representative strain in race 1, and only *OsSWEET11a* was significantly expressed in wild-type Kitaake infected with this strain. This finding was consistent with previous studies where PthXo1 was shown to be the only major TALE in PXO99^A^ (Eom et al. 2019). Similarly, only *OsSWEET14* was expressed significantly in cv. Kitaake rice infected with *Xoo* PXO86 (race 5); this result suggests that either PthXo3 or AvrXa7 was the major TALE in race 5 strains. When cv. Kitaake was infected with *Xoo* IXO221 (race 2), both *OsSWEET11a* and *OsSWEET14* were expressed. IXO221 also activated expression of *OsSWEET14* in MS13K and *OsSWEET11a* in MS34K, suggesting that there were two major TALEs in these strains, namely, PthXo1/ PthXo1* and PthXo3/ PthXo3*. When cv. Kitaake was infected with *Xoo* PXO61 (race 6), both *OsSWEET13* and *OsSWEET14* were expressed. PXO61 also activated.

*OsSWEET14* expression in MS13K and *OsSWEET13* in MS14K, suggesting the presence of two major TALEs in race 6 strains, namely PthXo2B/C and PthXo3/ PthXo3*. The race 3 strain, *Xoo* JS137-1, failed to activate expression of the three *OsSWEET* genes, suggesting that race 3 strains either lack major TALEs or perhaps harbor a variant of PthXo2B* which incompatible with cv. Kitaake. In contrast, all three *OsSWEET* genes were activated by *Xoo* strains in race 4, suggesting that these strains contain all three categories of major TALEs, namely, PthXo1(PthXo1*), PthXo2B/C and PthXo3(PthXo3*). This speculation was verified by phenotypic analysis (Fig. 2A) where all rice lines (except the triply-edited line MS134K) were susceptible to race 4 strains. Although we cannot accurately identify the presence or absence of PthXo2B*-like TALEs, our results indicate that *OsSWEET* gene expression correlates with the disease phenotype (resistant vs. susceptible) in inoculation tests, thus indicating that the identity of major TALE(s) of japonica rice can be predicted based on the inoculation phenotype of cv. Kitaake and its NILs.

### Prediction of Major TALEs in *Xoo* and Deployment of Rice Varieties

**Fig. 5:**
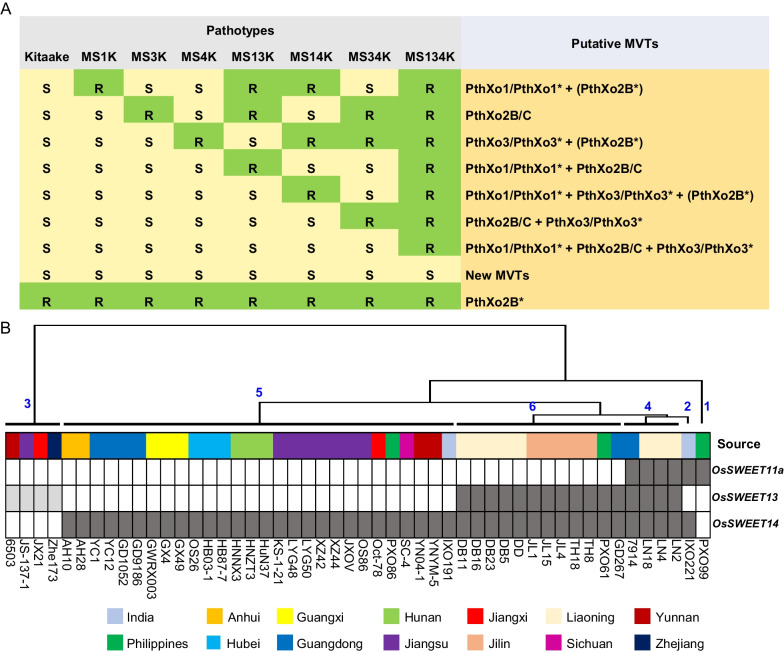
Major TALEs prediction and distribution of *Xoo* strains. **A** major TALE prediction table. The assignment of the major TALEs in *Xoo* strains was based on the inoculation phenotype observed in rice cv. Kitaake and the *OsSWEET* EBE edited lines. **B** Geographical distribution of *Xoo* strains and potential induction of *OsSWEET* genes. The heat map indicates whether *OsSWEET* genes were induced (gray rectangles) or not induced (white rectangles) by TALEs in *Xoo* strains. A parsimony tree (top) was generated based on NTSYS software and confirmed the resistant (R) and susceptible (S) phenotypes and clustered the strains into six clades (1–6, blue numbers)

A major TALE prediction table was developed based on the pathotypes observed when rice cv. Kitaake and the NILs were inoculated with different *Xoo* strains (Fig. 5A). The major TALEs in *Xoo* strains could be derived by analyzing the inoculation pathotypes on cv. Kitaake and the NILs, and the results help inform strategies for the effective distribution of disease-resistant varieties. For example, to identify suitable varieties of rice resistant to bacterial blight in different regions of China, we analyzed the geographic distribution of 50 *Xoo* strains and their probable *OsSWEET* targets. Based on results obtained from induction of *OsSWEET* genes, 45 *Xoo* strains contained major TALEs that could potentially activate *OsSWEET14* (Fig. 5B).

There were six and 16 *Xoo* strains that could possibly activate *OsSWEET11a* and *OsSWEET13*, respectively. Eight *Xoo* strains from Liaoning and five from Jilin activated *OsSWEET13* and *OsSWEET14*, and three Liaoning strains activated all three *OsSWEET* genes. Most *Xoo* strains (26/30) originating from southern China activated *OsSWEET14* but not *OsSWEET11a* or *OsSWEET13.* Our findings suggest that EBE editing of *OsSWEET14* could be used in southern China to combat *Xoo*, and EBE editing of *OsSWEET13* and *OsSWEET14* could be used in northeastern China.

### *OsSWEET11a *and *OsSWEET14* may Impair Rice Pollen Viability and Setting Rate

**Fig. 6:**
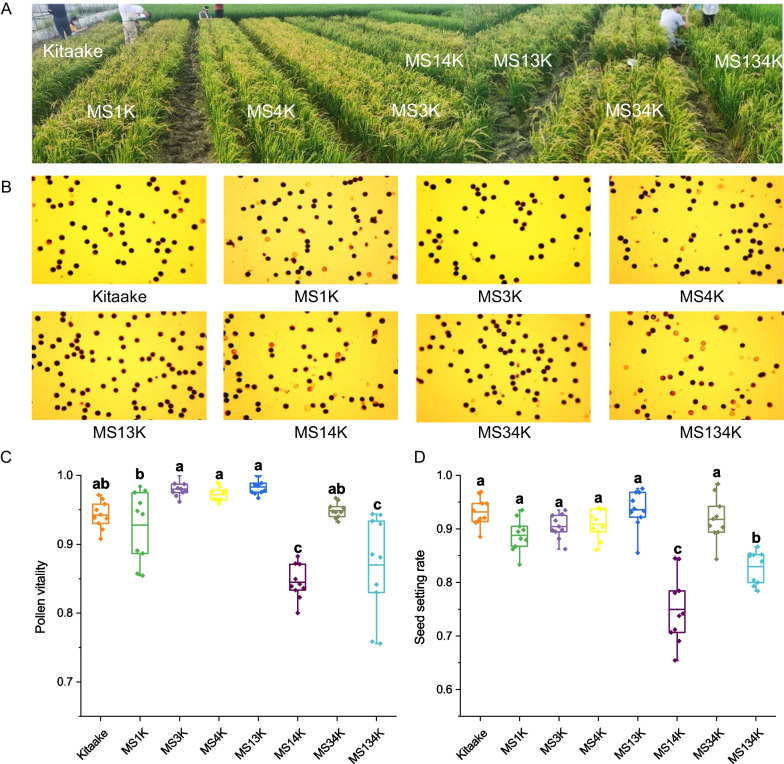
Pollen viability and setting rate of rice cv. Kitaake and EBE-edited lines. **A** Growth and development of Kitaake and homozygous, EBE-edited rice lines in the field. **B** Representative images of pollen viability tests from Kitaake and edited lines. Viable and sterile pollen grains stained dark and light yellow. **C**, **D** Statistical analysis of pollen viability (*n* = 10) and setting rates (*n* = 10) of Kitaake and EBE-edited lines. The percentage of pollen viability was calculated relative to total pollen counts obtained from eight microscopic images. The seed setting rate was calcuated as the percentage of solid grains relative to the total number of grains

The three *OsSWEET* genes are pluripotent and regulate susceptibility to *Xoo* in addition to participating in other physiological functions. For example, *OsSWEET11a* is presumably involved in another development, whereas *OsSWEET14* may function in grain filling (Chu et al. 2006; Sosso et al. 2015). Since EBE editing of *OsSWEET* genes could potentially impact other physiological functions in rice, we measured key agronomic traits in paddy experiments (Fig. 6A). Agronomic assessments and multivariant analysis of plant height, stem diameter, panicle numbers and grain weight indicated that most of the edited lines performed similarly to wild-type cv. Kitaake (Additional file 1: Figure S2). Compared with other lines, MS4K was taller, which implied that editing the *OsSWEET14* EBE may impact growth. The edited lines MS14K and MS134K had fewer grain numbers than cv. Kitaake, but MS14K had the highest grain weight. These results suggest that editing the EBEs in *OsSWEET11a* and *OsSWEET14* may reduce grain numbers but allow for a more concentrated distribution of energy among individual grains. The pollen viability of MS14K and MS134K was lower than the other rice lines, and the setting rates for these two lines correlated with pollen viability (Fig. 6B–D). Collectively, these results suggest that editing the EBEs may reduce the impact on rice growth and development, it still negatively affects pollen viability and setting rates, which warrants further investigation.

## Discussion

The coevolution of pathogens and host plants is dynamic and can lead to the emergence of novel pathogens with increased host range and diversity. A common situation is the introduction of new varieties with novel resistance genes, which exerts pressure on the pathogen population to evolve new virulence factors. Consequently, developing plant varieties with durable, broad-spectrum resistance is imperative. If pathogen strains and virulence factors are monitored in a timely fashion, the severity of disease can be mitigated. In this report, we developed a specific set of near-isogenic rice materials using CRISPR–Cas9 technology. To facilitate the application of this set of rice materials, we developed a major TALE prediction table based on the different pathotypes of cv. Kitaake and its NILs after inoculation with different *Xoo* strains (Fig. 5A). This table contains all observed pathotypes of cv. Kitaake and its NILs and can be used to deduce the putative major TALEs in *Xoo* strains. This set of differential rice lines include single, double and triple edits of EBE sequences in the promoters of three *OsSWEET* genes in rice cv. Kitaake. This set of rice materials can effectively predict the major TALEs in *Xoo* strains via plant inoculation and phenotypic monitoring and does not rely on genomic sequencing. The effectiveness of this set of materials was verified through genomic prediction and expression of susceptibility genes (*OsSWEET11a*, *OsSWEET13* and *OsSWEET14*). The collection of *Xoo* strains from different geographical regions, inoculation to edited rice lines and utilization of the TALE prediction table can provide insight into more intelligent breeding for bacterial blight resistance in rice.

The major resistance genes in rice interact with cognate avirulence genes in *Xoo*, and the compatibility of these interacting genes can confer disease resistance. However, the cultivation of rice varieties with a single resistance gene can be defeated by coevolving pathogen genes, and this often results in the defeat of resistance in a relatively short time. *Xoo* induces disease and host susceptibility by injecting TALEs into plant cells; this results in TALE binding to highly-conserved, cognate EBEs in the promoters of known *OsSWEET* genes. Traditional differential rice varieties for bacterial blight generally encode a series of resistance genes (Nugroho et al. 2022; Ogawa 1993); however, 47 *R* genes have been identified in rice for bacterial blight resistance (Lu et al. 2022), which makes it extremely difficult to construct NILs based on *R* genes. In contrast, susceptibility genes have limited diversity and only three *S* genes have been identified that are targeted by *Xoo* in nature; this suggests that effectors with new EBE-binding motifs may not evolve quickly (Oliva et al. 2019; Xu et al. 2019). The monitoring of bacterial blight races could be conducted more systematically based on *S* genes, which can detect *Xoo* strains with both single and multiple major TALEs. Based on the fact the *OsSWEET* genes have important physiological functions (Yang et al. 2018; Fei et al. 2021), we chose to edit the EBE sequences in the promoters rather than knocking out the *OsSWEET* genes to minimize the negative impacts on yields.

The five EBEs targeted by *Xoo* major TALEs include the following: PthXo1_EBE_ in the *OsSWEET11a* promoter; PthXo2_EBE_ and PthXo2-like_EBE_ in the *OsSWEET13* promoter of indica and japonica rice; and PthXo3_EBE_ and AvrXa7_EBE_ region in the promoter of *OsSWEET14* (This study focused on Asian strains, so TalC and TalF were not considered)*.* There are more than five variants of PthXo2 that bind to different EBE sequences (Oliva et al. 2019; Xu et al. 2019). Consequently, we selected the japonica rice cultivar cv. Kitaake, which has the highest proportion of EBE type in 3000 sequenced rice lines (Xu et al. 2019). When the 50 *Xoo* strains were screened in inoculation tests, 46 strains incite disease on cv. Kitaake and its EBE-edited lines. Another possible scenario is that an *Xoo* strain could elicit a susceptible phenotype on cv. Kitaake and all seven edited lines; this would suggest that a new major TALE has emerged that can induce an unidentified susceptibility.

Plant host evolution, environmental selection pressure and antagonistic interactions among *Xoo* strains provide sources of diversity in the *Xoo* population. It is quite possible that bacterial blight may be incited by more than one *Xoo* race in a given geographical area. Our results confirmed that regional convergence occurred among *Xoo* stains, and there were notable differences in the diversity of *Xoo* races from different geographical areas. For example, in the northeastern rice cultivation areas of China, most *Xoo* strains have major TALEs that can activate *OsSWEET13* and *OsSWEET14*, but some strains can activate *OsSWEET11a*, *OsSWEET13* and *OsSWEET14* simultaneously (e.g., *Xoo* LN2, LN4 and LN18). The phenomenon of regional differentiation is more common in the vast rice cultivation region in southern China, and most of the strains there only target the susceptibility gene *OsSWEET14*. In favorable environments with low selection pressure, the initial inoculum load in the environment develops and infects plants; moreover, genetic flow might play an important role in the dissemination of pathogens among different field populations. These results provide intriguing ideas for rice cultivation such as the development of rice cultivars with multiple mutations in EBEs, which may ultimately exhibit a form of broad-spectrum resistance. Furthermore, the expression of *OsSWEET11a*, *OsSWEET13*, and *OsSWEET14* in response to *Xoo* combined with geographical origin can suggest effective editing variants for rice cultivation.

Interestingly, genomic analysis has shown that *Xoo* LN18 does not encode PthXo1; however, recently published results from our laboratory suggest that other major TALEs reside in *Xoo* LN18 that can activate *OsSWEET11a* (Xu et al. 2023). Our results also showed that the fragment used in Southern blot hybridizations could not accurately detect the presence or absence of the major TALEs in *Xoo* strains. For example, our results showed that 94% of strains contained bands in the same position as PthXo1, but only 14% were able to induce *OsSWEET11a* expression based on leaf clipping. This is because Southern blot analysis can only detect the size of TALEs but cannot identify the TALE. These results suggest that the final pathotype determination still depended on plant materials.

Previous studies focusing on whether EBE modification affects rice yield and agronomic traits have been controversial (Li et al. 2020a, b; Ma et al. 2017). Our results showed that deletion of EBE sites from the *OsSWEET11a* and *OsSWEET14* promoters influenced the pollen viability and seed setting rates. Another study reported that single *OsSWEET11a* or *OsSWEET11b* mutants were fertile, but double mutants were infertile (Wu et al. 2022). In our study, single mutations in the *OsSWEET11a* and *OsSWEET14* EBEs mutants were fertile; however, double mutants were less fertile, suggesting a synergistic, negative effect on pollen viability. It is unclear whether the changes in agronomic traits are related to the editing position and/or the number of deleted bases. It is encouraging to see that EBE editing in the *OsSWEET13* promoter may have a positive impact on rice yield, which may reverse some of the negative effects caused by EBE-editing in *OsSWEET11a* and *OsSWEET14*.

In this study, editing the EBE regions of *S* gene promoters resulted in a relatively simple, rapid method for classifying pathogenic races of *Xoo.* This approach facilitates the identification of major pathogenic factors in *Xoo* strains that have not been sequenced and provides an opportunity for real-time surveillance of *Xoo* strains and deployment of resistant rice varieties. It is worth noting that, although there is not a single rice variety that can perfectly distinguish all PthXo2 variants on current technology measure, our study still provides an integrated technology or platform for combating bacterial blight disease in rice breeding and plantation. Moreover, because these rice materials balance yield and resistance, they can provide materials for scientific research and disease resistance breeding. Our future research efforts will extend to the identification of African *Xoo* strains, along with the development of homogeneous rice materials with more precise EBE editing. Due to the ongoing coevolution between pathogen effectors and rice, new major TALEs and target EBEs may be revealed in the future.

## Conclusion

In summary, the EBE-edited near-isogenic rice lines described in this study can be used as differential varieties to detect *Xoo* major virulence TALEs. The monitoring of *Xoo* races can be conducted more systematically using S genes as compared to R genes. Our set of these tracers can detect *Xoo* strains harboring both single and multiple MVTs and detect the uncertain virulence TALEs of genomic prediction. Furthermore, the EBE-edited lines can guarantee an optimum balance in yield and resistance and provide clues on what rice cultivars are needed to combat the ongoing evolution of *Xoo* in the field.

## Materials and Methods

### Bacterial Strains, Plasmids and Media

The bacterial strains and plasmids used in this study are listed in Additional file 1: Table S7. *Escherichia coli* strains were grown in Luria Bertani (LB) medium supplemented with the appropriate antibiotics at 37 °C, and *Agrobacterium tumefaciens* strains were cultured in LB medium containing rifampicin at 28 °C. Forty-five of the *Xoo* strains used in this study were isolated from rice cultivated in 11 provinces in China, whereas the other five *Xoo* strains were collected from the Philippines and India (Additional file 1: Table S2). All *Xoo* strains were grown in nutrient broth (NB) at 28℃. The final concentrations of antibiotics were as follows: rifampicin, 75 μg ml^−1^; kanamycin, 25 μg ml^−1^; and spectinomycin, 100 μg ml^−1^.

### Generation of Rice Mutant Lines with CRISPR/Cas9 Technology

Rice cv. Kitaake was used for CRISPR/Cas9-mediated genome editing of *OsSWEET11a*, *OsSWEET13* and *OsSWEET14* as described (Zhou et al. 2014). Briefly, target sequences were selected within the promoter regions of *OsSWEET11a*, *OsSWEET13* and *OsSWEET14*, and sgRNAs were designed with CRISPR MultiTargeter (http://www.multicrispr.net/index.html). The specificity of target sequences was verified using the BLAST function in NCBI (http://blast.ncbi.nlm.nih.gov/Blast.cgi, Additional file 1: Table S8). The target sequences were synthesized by Tsingke Biotechnology Co., Ltd. (Beijing, China), and double-stranded oligonucleotide DNA (dsOligo) was formed after annealing. The dsOligos corresponding to the three *OsSWEET* EBEs were individually inserted into *Bsa*I- or *Btg*ZI-digested pENTR4-gRNA4 to form vectors designed to mutant a single EBE in each of the three *OsSWEET* promoter regions (Additional file 1: Figure S3). Another dsOligo targeting a second *OsSWEET* EBE was inserted into an intermediate vector containing a different *OsSWEET* EBE to form a construct designed to mutant two EBEs simultaneously (Additional file 1: Figure S4), and the triple mutant rice line was obtained by a single mutation in the *OsSWEET13* EBE of MS14K (Xu et al. 2019). Constructs containing single and double edits in the *OsSWEET* EBEs were transferred into pBY02-Cas9 using Gateway LR Clonase (Thermo Fisher Scientific). The guide RNA (gRNA) and CRISPR/Cas9 constructs were verified by *Bam*HI digestion (Additional file 1: Figure S5) and Sanger sequencing (data not shown). pBY02-Cas9 constructs containing mutant forms of *OsSWEET* EBEs were transferred into Kitaake callus by *Agrobacterium*-mediated transformation (Biorun, Wuhan, China). Individual rice transformants were selected and genomic DNA was isolated; PCR amplification and Sanger sequencing of the target region was used to select mutant lines MS1K, MS3K, MS4K, MS13K, MS34K, and MS134K in generations T0 to T3 (Additional file 1: Additional file 1: Table S1). The primers utilized to confirm the mutants are listed in Additional file 1: Table S9.

### Plant Material, Growth Conditions and Pathogen Inoculation Assays

Seeds of rice cv. Kitaake and mutant lines were soaked in water for 48 h and planted in the field as two-week-old seedlings. Rice plants were randomly planted in fields with consistent monitoring at the Zhuanghang Comprehensive Experiment Station and Shanghai Jiao Tong University in Shanghai in the summers of 2021, 2022, and 2023. Leaves of eight-week-old rice plants were dissected with scissors previously immersed in suspensions of *Xoo* strains, and lesion lengths were measured 14 d after inoculation. Lesion length measurements ≤ 3 cm were scored as resistant (R), and ≥ 3 cm as susceptible (S). Disease assays were performed at least three times.

### Evaluation of Rice Mutant Lines for Agronomic Traits and Pollen Viability

Plant height, stem diameter, panicle numbers, grain numbers and grain weight were assessed in cv. Kitaake and mutant lines under paddy conditions using a randomized block design with three replications. Setting rates (grain numbers/panicle numbers) were calculated, and pollen viability was evaluated as previously described (Chhun et al. 2007). Briefly, six anthers from cv. Kitaake and mutant lines were removed and crushed into powder; pollen grains were then stained with 10 μl I_2_-KI solution (1% I_2_, 3% KI) and observed using a light microscope. Fertile (dark) and infertile (yellow) pollen grains were counted, and the percentage of pollen viability was calculated in six microscopic images.

### Southern Blotting

Genomic DNA of 50 *Xoo* strains was extracted using the HiPure Bacterial DNA Extraction Kit (Magen, Guangzhou, China). DNA samples (50 µl) were digested with *Bam*HI for 4 h at 37℃. The resulting DNA fragments were separated in 1.3% agarose gels at 80 V for 22 h and transferred to Immobilon-Ny + membranes (Millipore, USA). A hybridization probe was made from a digoxigenin (DIG)-labeled 2892-bp fragment derived from an *Sph*I-digest containing the repetitive sequence of *pthXo1* (GenBank: AY495676). The Detection Starter Kit I (Roche, Switzerland) was used to visualize hybridizing fragments according to the manufacturer’s instructions. The TALE-free strain *Xoo* PH containing an introduced copy of *pthXo1*, *pthXo2* or *avrXa7* (Additional file 1: Table S6) was used to locate the major *tal* genes involved in virulence.

### RNA Isolation and qRT–PCR

Fourteen days after inoculation, total RNA was isolated (TRIzol, Invitrogen, USA) by sampling the intersection of diseased and healthy tissues in rice leaves. Total RNA was used for cDNA synthesis with the EasyScript gDNA Removal and cDNA Synthesis Supermix (Transgen Biotech). Resulting cDNAs were used for qRT-PCR, which was performed using the TransStart Tip Green qPCR SuperMix (Transgen Biotech) and the ABI7500 Real-Time PCR System (Applied Biosystems, USA). The 2^−ΔΔCt^ method was used for calculating the relative expression of *OsSWEET11a*, *OsSWEET13* and *OsSWEET14*, and the primers used are listed in Additional file 1: Table S9. Gene expression was normalized using *OsActin*, and qRT–PCR experiments were repeated at least three times.

### Statistical Analysis

TALEs were annotated for nine *Xoo* strains with known genomic sequences using AnnoTALE software (Grau et al. 2016). The nine strains and NCBI accession numbers were as follows: *Xoo* PXO61, accession no. NZ_CP033187.3; *Xoo* PXO86, NZ_CP007166.1; *Xoo* PXO99^A^, NC_010717.2; *Xoo* IXO221, NZ_CP059591.1; *Xoo* AH28, NZ_CP074076.1; *Xoo* HuN37, NZ_CP031456.1; *Xoo* LN4, CP045452.1; *Xoo* LN18, CP045238.1; and *Xoo* JS137-1, unpublished). Pathotype assignment and clustering were conducted using the unweighted pair group mean algorithm (UPGMA) implemented in NTSYS software (Rohlf et al. 2000), and boxplots were generated using ORIGIN 2018. One-way analysis of variance (ANOVA) and Tukey’s honestly significant difference test was used for all measurements, and results were considered significant at *P* < 0.05.

### Supplementary Information

**Additional file 1.** Supplementary Figures and Tables.

## Data Availability

The datasets used and analyzed in the current study are available from the corresponding author on request. Rice materials will be available for non-for-profit research by material transfer agreements issued by the corresponding author.

## Abbreviations

*Xoo*: *Xanthomonas oryzae* pv. *oryzae*
TALE: Transcription activator-like effectors
S gene: Susceptibility gene
R gene: Resistant gene
EBE: Effector-binding element
NILs: Near-isogenic lines
PTI: Pattern-triggered immunity
ETI: Effector-triggered immunity
ETS: Effector-triggered susceptibility
SWEET: Sugars will eventually be exported transporter
BB: Bacterial leaf blight
RVD: Repeat variable di-residues

## References

[CR1] Antony G, Zhou J, Huang S, Li T, Liu B, White F, Yang B (2010). Rice xa13 recessive resistance to bacterial blight is defeated by induction of the disease susceptibility gene Os-11N3. Plant Cell.

[CR2] Boch J, Bonas U (2010). Xanthomonas AvrBs3 family-type III effectors: discovery and function. Annu Rev Phytopathol.

[CR3] Boch J, Scholze H, Schornack S, Landgraf A, Hahn S, Kay S, Lahaye T, Nickstadt A, Bonas U (2009). Breaking the code of DNA binding specificity of TAL-type III effectors. Science.

[CR4] Chen L-Q, Hou B-H, Lalonde S, Takanaga H, Hartung ML, Qu X-Q, Guo W-J, Kim J-G, Underwood W, Chaudhuri B, Chermak D, Antony G, White FF, Somerville SC, Mudgett MB, Frommer WB (2010). Sugar transporters for intercellular exchange and nutrition of pathogens. Nature.

[CR5] Chen X, Liu P, Mei L, He X, Chen L, Liu H, Shen S, Ji Z, Zheng X, Zhang Y, Gao Z, Zeng D, Qian Q, Ma B (2021). Xa7, a new executor R gene that confers durable and broad-spectrum resistance to bacterial blight disease in rice. Plant Commun.

[CR6] Chhun T, Aya K, Asano K, Yamamoto E, Morinaka Y, Watanabe M, Kitano H, Ashikari M, Ueguchi-Tanaka MM (2007). Gibberellin regulates pollen viability and pollen tube growth in rice. Plant Cell.

[CR7] Chu Z, Yuan M, Yao J, Ge X, Yuan B, Xu C, Li X, Fu B, Li Z, Bennetzen JL, Zhang Q, Wang S (2006). Promoter mutations of an essential gene for pollen development result in disease resistance in rice. Genes Dev.

[CR8] Eom J-S, Luo D, Atienza-Grande G, Yang J, Ji C, Thi Luu V, Huguet-Tapia JC, Char SN, Liu B, Nguyen H, Schmidt SM, Szurek B, Vera Cruz C, White FF, Oliva R, Yang B, Frommer WB (2019). Diagnostic kit for rice blight resistance. Nat Biotechnol.

[CR9] Fei H, Yang Z, Lu Q, Wen X, Zhang Y, Zhang A, Lu C (2021). OsSWEET14 cooperates with OsSWEET11 to contribute to grain filling in rice. Plant Sci.

[CR10] Grau J, Reschke M, Erkes A, Streubel J, Morgan RD, Wilson GG, Koebnik R, Boch J (2016). AnnoTALE: bioinformatics tools for identification, annotation and nomenclature of TALEs from Xanthomonas genomic sequences. Sci Rep.

[CR11] Gupta PK (2020). SWEET genes for disease resistance in plants. Trends Genet.

[CR12] Huang F, He N, Yu M, Li D, Yang D (2023). Identification and fine mapping of a new bacterial blight resistance gene, Xa43(t), in Zhangpu wild rice (*Oryza rufipogon*). Plant Biol.

[CR13] Ji Z, Zakria M, Zou L, Xiong L, Chen G (2014). Genetic diversity of transcriptional activator-like effector genes in Chinese isolates of *Xanthomonas oryzae* pv. oryzicola. Phytopathology.

[CR14] Ji Z, Ji C, Liu B, Zou L, Chen G, Yang B (2016). Interfering TAL effectors of *Xanthomonas oryzae* neutralize R-gene-mediated plant disease resistance. Nat Commun.

[CR15] Ji C, Ji Z, Liu B, Cheng H, Liu H, Liu S, Yang B, Chen G (2020). Xa1 Allelic R genes activate rice blight resistance suppressed by interfering TAL effectors. Plant Commun.

[CR16] Jiang N, Yan J, Liang Y, Shi Y, Peng J (2020). Resistance genes and their interactions with bacterial blight/leaf streak pathogens (*Xanthomonas oryzae*) in Rice (*Oryza sativa* L.)—an updated review. Rice.

[CR17] Jones JD, Dangl JL (2006). The plant immune system. Nature.

[CR18] Khojasteh M, Shah SMA, Haq F, Xu X, Taghavi SM, Osdaghi E, Chen G (2020). Transcription activator-like effectors diversity in Iranian strains of *Xanthomonas translucens*. Phytopathology.

[CR19] Li C, Li W, Zhou Z, Chen H, Xie C, Lin Y (2020). A new rice breeding method: CRISPR/Cas9 system editing of the Xa13 promoter to cultivate transgene-free bacterial blight-resistant rice. Plant Biotechnol J.

[CR20] Li W, Deng Y, Ning Y, He Z, Wang GL (2020). Exploiting broad-spectrum disease resistance in crops: from molecular dissection to breeding. Annu Rev Plant Biol.

[CR21] Lu Y, Zhong Q, Xiao S, Wang B, Ke X, Zhang Y, Yin F, Zhang D, Jiang C, Liu L, Li J, Yu T, Wang L, Cheng Z, Chen L (2022). A new NLR disease resistance gene Xa47 confers durable and broad-spectrum resistance to bacterial blight in rice (Original Research). Front Plant Sci.

[CR22] Ma L, Zhang D, Miao Q, Yang J, Xuan Y, Hu Y (2017). Essential role of sugar transporter OsSWEET11 during the early stage of rice grain filling. Plant Cell Physiol.

[CR23] Mondal KK, Meena BR, Junaid A, Verma G, Mani C, Majumder D, Khicher M, Kumar S, Banik S (2014). Pathotyping and genetic screening of type III effectors in Indian strains of *Xanthomonas oryzae* pv. oryzae causing bacterial leaf blight of rice. Physiol Mol Plant Pathol.

[CR24] Moscou MJ, Bogdanove AJ (2009). A simple cipher governs DNA recognition by TAL effectors. Science.

[CR25] Ngou BPM, Ding P, Jones JDG (2022). Thirty years of resistance: Zig–zag through the plant immune system. Plant Cell.

[CR26] Nugroho C, Cumagun CJR, Oliva R (2022). Diversity of *Xanthomonas oryzae* pv. Oryzae on susceptible and resistant rice lines in bacterial blight hot spot areas of the Philippines. Eur. J. Plant Pathol..

[CR27] Ogawa T (1993). Methods and strategy for monitoring race distribution and identification of resistance genes to bacterial leaf blight (*Xanthomonas campestris* pv. oryzae) in rice. JARQ.

[CR28] Oliva R, Ji C, Atienza-Grande G, Huguet-Tapia JC, Perez-Quintero A, Li T, Eom J-S, Li C, Nguyen H, Liu B, Auguy F, Sciallano C, Luu VT, Dossa GS, Cunnac S, Schmidt SM, Slamet-Loedin IH, Vera Cruz C, Szurek B, Frommer WB, White FF, Yang B (2019). Broad-spectrum resistance to bacterial blight in rice using genome editing. Nat Biotechnol.

[CR29] Rohlf F (2000) NTSYS-pc, Numerical Taxonomy and Multivariate Analysis System, version 2.1.

[CR30] Sosso D, Luo D, Li QB, Sasse J, Yang J, Gendrot G, Suzuki M, Koch KE, McCarty DR, Chourey PS, Rogowsky PM, Ross-Ibarra J, Yang B, Frommer WB (2015). Seed filling in domesticated maize and rice depends on SWEET-mediated hexose transport. Nat Genet.

[CR31] Streubel J, Pesce C, Hutin M, Koebnik R, Boch J, Szurek B (2013). Five phylogenetically close rice SWEET genes confer TAL effector-mediated susceptibility to *Xanthomonas oryzae* pv. oryzae. New Phytol.

[CR32] Timilsina S, Potnis N, Newberry EA, Liyanapathiranage P, Iruegas-Bocardo F, White FF, Goss EM, Jones JB (2020). Xanthomonas diversity, virulence and plant–pathogen interactions. Nat Rev Microbiol.

[CR33] Tran TT, Pérez-Quintero AL, Issa W, Carpenter S, Yu Y, Wang L, Leach JE, Valérie V, Sébastien C, Bogdanove AJ (2018). Functional analysis of African *Xanthomonas oryzae* pv. oryzae TALomes reveals a new susceptibility gene in bacterial leaf blight of rice. Plos Pathogens.

[CR34] Wu LB, Eom JS, Isoda R, Li C, Char SN, Luo D, Van SL, Nakamura M, Yang B, Frommer WB (2022). OsSWEET11b, a potential sixth leaf blight susceptibility gene involved in sugar transport-dependent male fertility. New Phytol.

[CR35] Xu Z, Xu X, Gong Q, Li Z, Li Y, Wang S, Yang Y, Ma W, Liu L, Zhu B, Zou L, Chen G (2019). Engineering broad-spectrum bacterial blight resistance by simultaneously disrupting variable TALE-binding elements of multiple susceptibility genes in rice. Mol Plant.

[CR36] Xu Z, Xu X, Wang Y, Liu L, Li Y, Yang Y, Liu L, Zou L, Chen G (2022). A varied AvrXa23-like TALE enables the bacterial blight pathogen to avoid being trapped by Xa23 resistance gene in rice. J Adv Res.

[CR37] Xu Z, Xu X, Li Y, Liu L, Wang Q, Wang Y, Wang Y, Yan J, Cheng G, Zou L, Zhu B, Chen G (2023). Tal6b/AvrXa27A, a Hidden TALE targeting both the susceptibility gene OsSWEET11a and the resistance gene Xa27 in rice. Plant Commun.

[CR38] Yang B, White FF (2004). Diverse members of the AvrBs3/PthA family of type III effectors are major virulence determinants in bacterial blight disease of rice. Mol Plant Microbe Interact.

[CR39] Yang B, Sugio A, White F (2006). Os8N3 is a host disease-susceptibility gene for bacterial blight of rice. Proc Natl Acad Sci.

[CR40] Yang J, Luo D, Yang B, Frommer WB, Eom J-S (2018). SWEET11 and 15 as key players in seed filling in rice. New Phytol.

[CR41] Yuan M, Wang S (2013). Rice MtN3/Saliva/SWEET family genes and their homologs in cellular organisms. Mol Plant.

[CR42] Yuan M, Jiang Z, Bi G, Nomura K, Liu M, Wang Y, Cai B, Zhou J-M, He SY, Xin X-F (2021). Pattern-recognition receptors are required for NLR-mediated plant immunity. Nature.

[CR43] Zhang B, Zhang H, Li F, Ouyang Y, Yuan M, Li X, Xiao J, Wang S (2020). Multiple alleles encoding atypical NLRs with unique central tandem repeats in rice confer resistance to *Xanthomonas oryzae* pv. oryzae. Plant Commun.

[CR44] Zhou H, Bo L, Weeks DP, Spalding MH, Bing Y (2014). Large chromosomal deletions and heritable small genetic changes induced by CRISPR/Cas9 in rice. Nucleic Acids Res.

[CR45] Zhou J, Peng Z, Long J, Sosso D, Liu B, Eom JS, Huang S, Liu S, Vera Cruz C, Frommer WB, White FF, Yang B (2015). Gene targeting by the TAL effector PthXo2 reveals cryptic resistance gene for bacterial blight of rice. Plant J.

