# Supplementary material for: Constructed Rice Tracers Identify the Major Virulent Transcription Activator-Like Effectors of the Bacterial Leaf Blight Pathogen
Source: Rice (N Y). 2024 Apr 24;17:30. doi: 10.1186/s12284-024-00704-0 (PMC11043257; doi:10.1186/s12284-024-00704-0)
Supplement: Supplementary file 1 — Additional file 1. Supplementary Figures and Tables. [file 12284_2024_704_MOESM1_ESM.docx]

**Supplemental Information**

Article title: Constructed rice-tracers identify the major virulent transcription activator-like effectors of bacterial leaf blight pathogen

Authors: Linlin Liu, Ying Li, Qi Wang, Xiameng Xu, Jiali Yan, Yong Wang, Yijie Wang, Syed Mashab Ali Shah, Yongzheng Peng, Zhangfei Zhu, Zhengyin Xu, and Gongyou Chen

The following Supporting Information is available for this article:


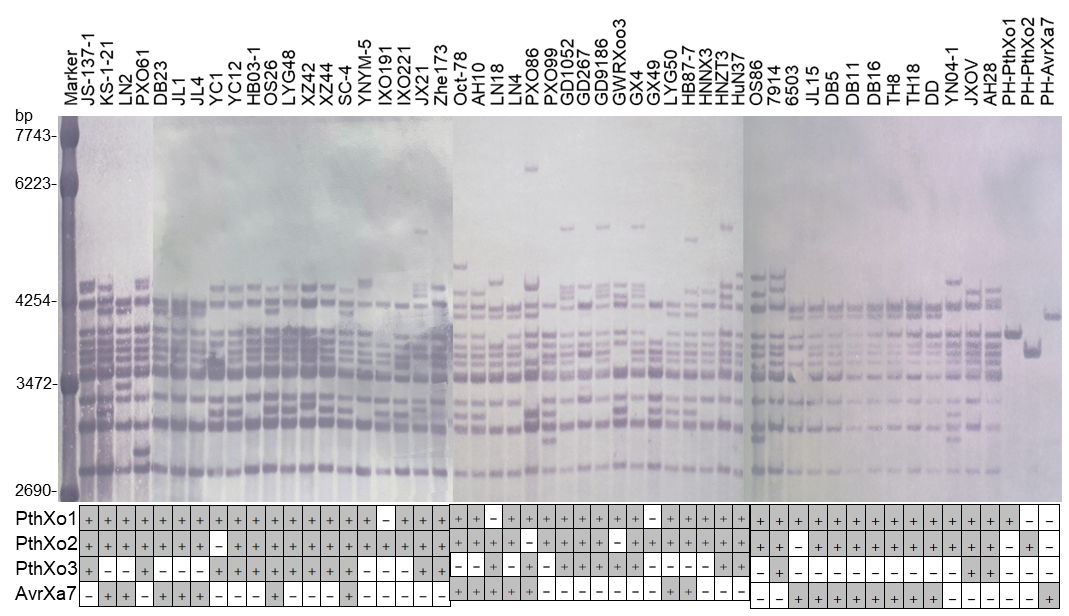


**Supplemental Figure 1 Southern blotting of 50 Xoo strains.** Genomic DNA was digested with BamHI, transferred to nylon membranes, and hybridized with the SphI fragment from pthXo1. The location of pthXo1, pthXo2 and avrXa7 were determined by comparing with Xoo strain PH containing pthXo1, pthXo2 or avrXa7, respectively. The (+) and (-) symbols indicate the presence and absence of pthXo1, pthXo2, pthXo3 or avrXa7, respectively.

**
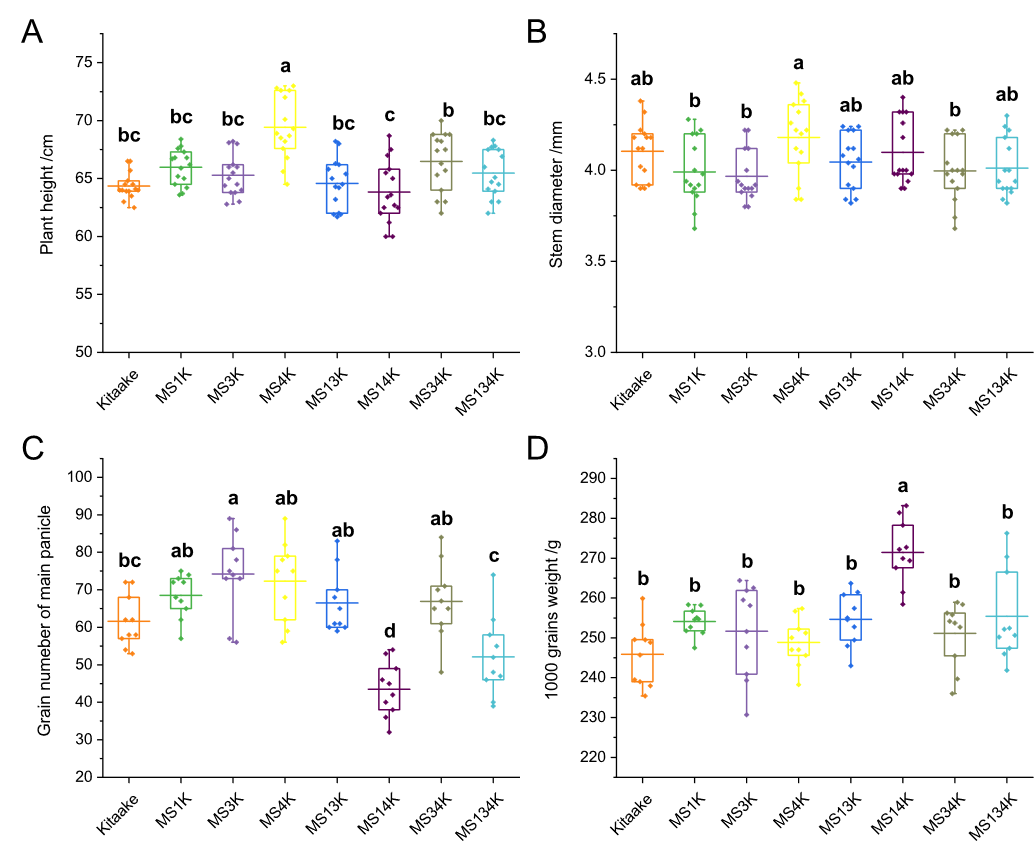
**

**Supplemental Figure 2 Agronomic traits in rice cv. Kitaake and seven EBE-edited lines.** Experiments in paddy fields were conducted using a randomized complete block design with three replicates. **a**, Plant height (*n*=15). **b,** Stem diameters at the third internode (*n*=15). **c,** Grain numbers per main panicle (*n*=10). **d,** 1000-grain weight (*n*=10). Boxplots labeled with different letters indicate significant differences among mutants (*P*<0.05). Trials were conducted in a single season.

**
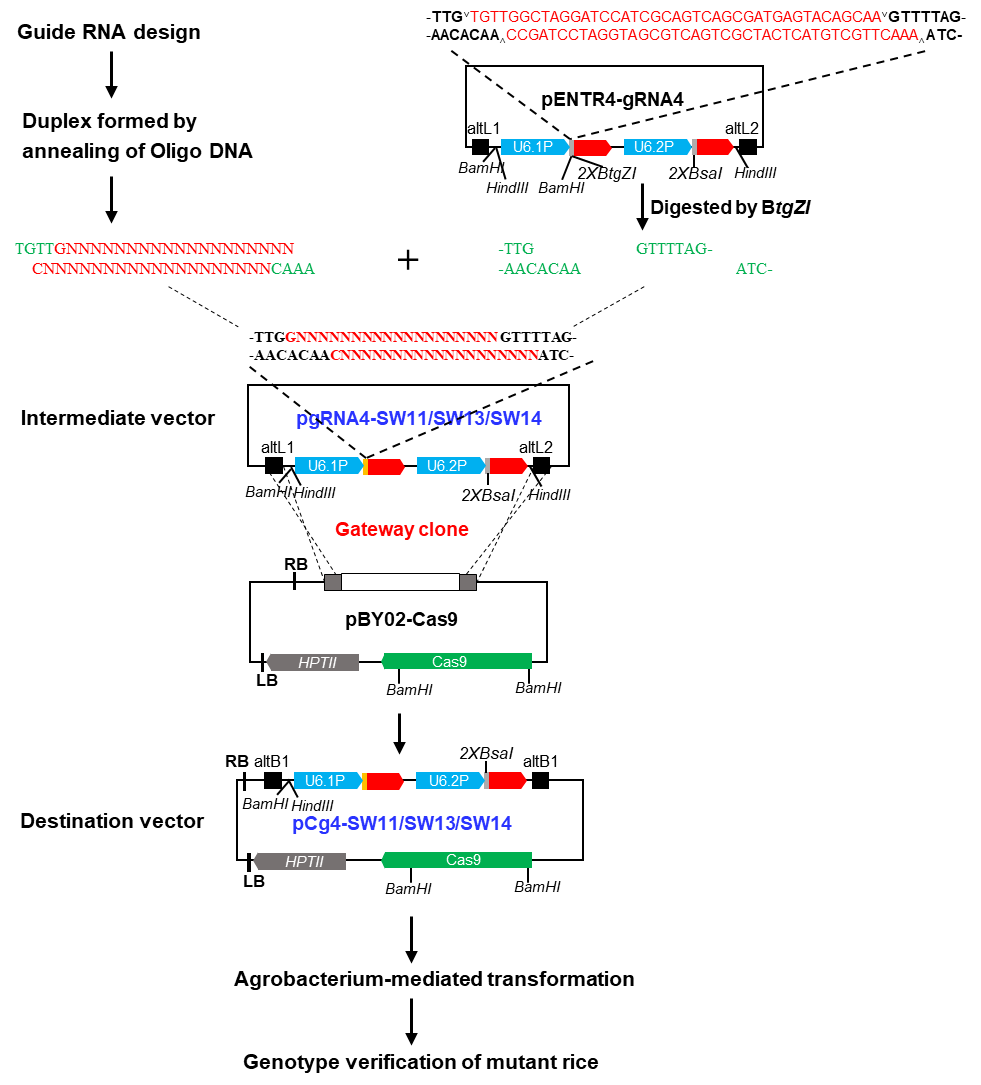
**

**Supplemental Figure 3 Vector construction for editing single EBEs using the CRISPR/Cas9 system.** Target sequences were selected and designed with CRISPR MultiTargeter. Single stranded primers were synthesized, and the dsOligo DNA was formed by annealing. The dsOligo DNAs were ligated with *Btg*ZI-digested pENTR4-gRNA4 to form intermediate vectors and then inserted into pBY02-Cas9 by Gateway cloning. Destination vectors containing mutant forms of *OsSWEET* EBEs in pBY02-Cas9 were transferred into Kitaake callus by *Agrobacterium*-mediated transformation.

**
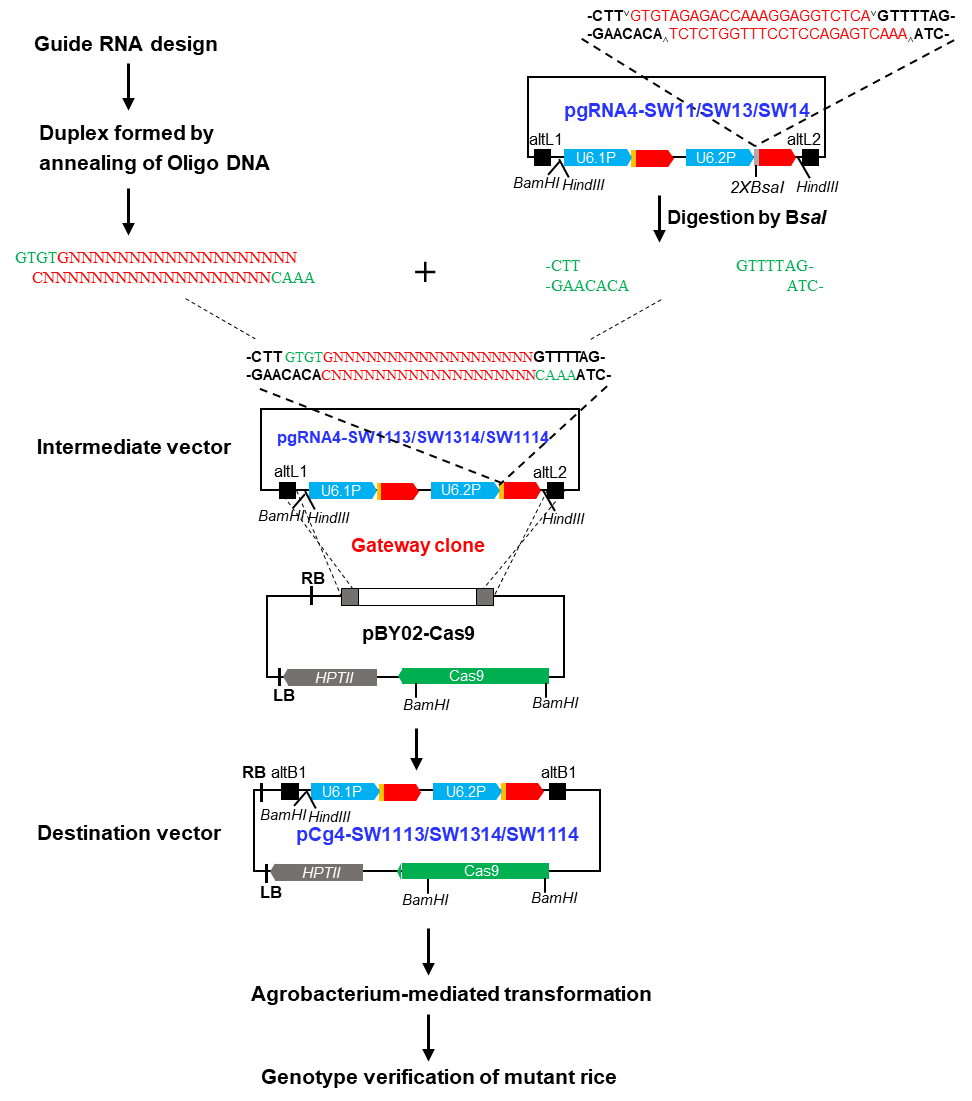
**

**Supplemental Figure 4 Vector construction for editing two EBEs simultaneously using the CRISPR/Cas9 system.** Target sequences for editing a second EBE were synthesized, and the dsOligo DNAs were formed by annealing. The dsOligo DNA was ligated with *Bsa*I-digested pENTR4-SWXX (containing a single edit in one of the *OsSWEET* EBEs) to form an intermediate vector. The intermediate vectors were then inserted into pBY02-Cas9 vector by Gateway cloning. Destination vectors containing two mutant forms of *OsSWEET* EBEs in pBY02-Cas9 were transferred into Kitaake callus by *Agrobacterium*-mediated transformation.

**
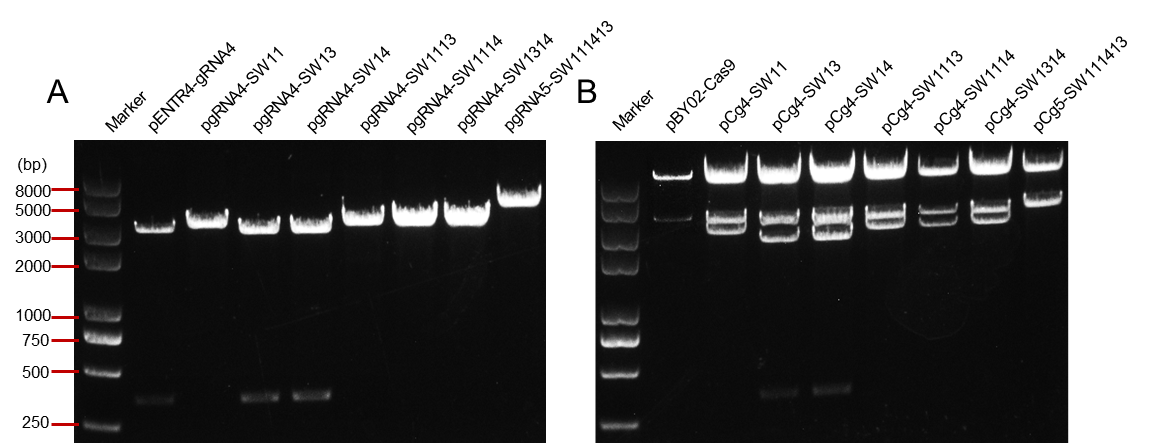
**

**Supplemental Figure 5 Construction of edited rice lines. a**, Verification of guide RNA constructs used in edited lines MS1K (pgRNA4-SW11), MS3K (pgRN4A-SW13), MS4K (pgRNA4-SW14), MS13K (pgRNA4-SW1113), MS14K (pgRNA4-SW14K), MS34K (pgRNA4-SW1314) and MS134K (pgRNA5-SW111413). There are two *Bam*HI digestion sites in pENTR4-gRNA4, and one of these overlaps with *Btg*ZI. When the target sequence is inserted into the *Bsa*I site of pENTR4- gRNA4, there will be two bands after digestion with *Bam*HI (see pgRNA4-SW13 and pgRNA4-SW14). When the target sequence is inserted into the *Btg*ZI site, there will only be one band after digestion with *Bam*HI (see pgRNA4-SW11, pgRNA4-SW1113, pgRNA4-SW1314 and pgRNA5-SW111413). **b**, Verification of CRISPR/Cas9 constructs used for editing MS1K (pCg4-SW11), MS3K (pCg4-SW13), MS4K (pCg4-SW14), MS13K (pCg4-SW1113), MS14K (pCg4-SW1114), MS34K (pCg4-SW1314) and MS134K (pCg5-SW111413)*.* In addition to the two *Bam*HI digestion sites in pENTR4-gRNA4, pBY02-Cas9 also has two *Bam*HI sites.

**Supplemental Table 1. EBE sequences in three *OsSWEET* genes of rice cv. Kitaake and mutants.**

| Rice lines | Generation^1^ | *OsSWEET11p*^2^ | *OsSWEET13p*^3^ | *OsSWEET14p* |
| --- | --- | --- | --- | --- |
| Kitaake |  | GCATCTCCCCCTACTGTACACCACCAA | ATAAAGCACCACAACTCCCTT | ATAAACCCCCTCCAACCAGGTGCTAAG |
| MS3K | T0 | GCATCTCCCCCTACTGTACACCACCAA | A=====CACCACAACTCCCTT(-5) | ATAAACCCCCTCCAACCAGGTGCTAAG |
| MS4K | T0 | GCATCTCCCCCTACTGTACACCACCAA | ATAAAGCACCACAACTCCCTT | ATAAAC=====CCAACCAGGTGCTAAG(-5) |
| MS13K | T0 | GCATCTCCCCCTA====CCACCACCAA（-4） | ANNNNNNNNNNNNNNNNNNNN | ATAAACCCCCTCCAACCAGGTGCTAAG |
|  | T1 | GCATCTCCCCCTA====CCACCACCAA（-4） | ATAA==CACCACAACTCCCTT(-2) | ATAAACCCCCTCCAACCAGGTGCTAAG |
| MS34K | T0 | GCATCTCCCCCTACTGTACACCACCAA | A=====CACCACAACTCCCTT（-5） | ATAAACCCCNNNNNNNNNNNNNNNNNN |
|  | T1 | GCATCTCCCCCTACTGTACACCACCAA | A=====CACCACAACTCCCTT（-5） | ATAAACCCCC=======AGGTGCTAAG(-7) |
| MS134K | T0-16 | GCATCTCCCCCTACT=====CCACCAA(-5) | ATAAAGCACCACNNNNNNNNN | ATATAAACCCC=T====CCAGGTGCTAAG(-5) |
|  | T1-16 | GCATCTCCCCCTACT=====CCACCAA(-5) | ATAAAGCACCACNNNNNNNNN | ATATAAACCCC=T====CCAGGTGCTAAG(-5) |
|  | T2-16 | GCATCTCCCCCTACT=====CCACCAA(-5) | ATAAAGCACCACNNNNNNNNN | ATATAAACCCC=T====CCAGGTGCTAAG(-5) |
|  | T3-16 | GCATCTCCCCCTACT=====CCACCAA(-5) | ATAAAGCACCAC=====CCTT(-5) | ATATAAACCCC=T====CCAGGTGCTAAG(-5) |

^1^Generations chosen for further study are shaded in green.

^2^ “=”, indicates homozygosity at the deletion site.

^3^, Nucleotides (“N”) in red font indicate the site was heterozygosis.

**Supplemental Table 2 *Xoo* strains used in this study.**

| **Strains** | **Origin** | **Reference/Source** | **Accession number** |
| --- | --- | --- | --- |
| AH10 | Anhui, China | This lab | / |
| AH28 | Anhui, China | (Xu et al., 2022) | NZ_CP074076.1 |
| 7914 | Guangdong, China | This lab | / |
| GD1052 | Guangdong, China | This lab | / |
| GD267 | Guangdong, China | This lab | / |
| GD9186 | Guangdong, China | This lab | / |
| YC1 | Guangdong, China | This lab | / |
| YC12 | Guangdong, China | This lab | / |
| GWRXoo3 | Guangxi, China | This lab | / |
| GX4 | Guangxi, China | This lab | / |
| GX49 | Guangxi, China | This lab | / |
| HB03-01 | Hubei, China | This lab | / |
| HB87-7 | Hubei, China | This lab | / |
| OS26 | Hubei, China | This lab | / |
| HNNX3 | Hunan, China | This lab | / |
| HNZT3 | Hunan, China | This lab | / |
| HuN37 | Hunan, China | This lab | NZ_CP031456.1 |
| JS137-1 | Jiangsu, China | This lab | unpublished |
| JXOV | Jiangsu, China | This lab | / |
| KS-1-21 | Jiangsu, China | This lab | / |
| LYG48 | Jiangsu, China | This lab | / |
| LYG50 | Jiangsu, China | This lab | / |
| OS86 | Jiangsu, China | This lab | / |
| XZ42 | Jiangsu, China | This lab | / |
| XZ44 | Jiangsu, China | This lab | / |
| Oct-78 | Jiangxi, China | This lab | / |
| JX21 | Jiangxi, China | This lab | / |
| JL1 | Jilin, China | This lab | / |
| JL15 | Jilin, China | This lab | / |
| JL4 | Jilin, China | This lab | / |
| TH18 | Jilin, China | This lab | / |
| TH8 | Jilin, China | This lab | / |
| DB11 | Liaoning, China | This lab | / |
| DB16 | Liaoning, China | This lab | / |
| DB23 | Liaoning, China | This lab | / |
| DB5 | Liaoning, China | This lab | / |
| DD | Liaoning, China | This lab | / |
| LN18 | Liaoning, China | (Xu et al., 2019) | CP045238.1 |
| LN2 | Liaoning, China | This lab | / |
| LN4 | Liaoning, China | (Xu et al., 2020) | CP045452.1 |
| SC-4 | Sichuan, China | This lab | / |
| 6503 | Yunnan, China | This lab | / |
| YN04-1 | Yunnan, China | This lab | / |
| YNYM-5 | Yunnan, China | This lab | / |
| Zhe173 | Zhejiang, China | This lab | / |
| IXO191 | India | This lab | / |
| IXO221 | India | This lab | NZ_CP059591.1 |
| PXO61 | Philippines | (Yang and White, 2004) | NZ_CP021789.1 |
| PXO86 | Philippines | (Hopkins et al., 1992) | NZ_CP007166.1 |
| PXO99A | Philippines | (Hopkins et al., 1992) | NC_010717.2 |
| PH | This lab | (Ji et al., 2016) |  |
| PH(*pthXo1*) | This lab | (Ji et al., 2016) | / |
| PH(*pthXo2*) | This lab | (Xu et al., 2019) | / |
| PH(*avrXa7*) | This lab | (Ji et al., 2016) | / |

**Supplemental Table 3 Lesion lengths (cm) of *Xoo* strains on rice cv. Kitaake and EBE-edited rice lines.**

| Strains | Kitaake | MS1K | MS3K | MS4K | MS13K | MS14K | MS34K | MS134K |
| --- | --- | --- | --- | --- | --- | --- | --- | --- |
| Oct-78 | 13.19±1.44 | 7.27±1.50 | 8.27±1.35 | 1.88±0.87 | 8.54±2.66 | 1.15±0.49 | 1.31±0.64 | 1.62±0.76 |
| AH10 | 11.65±2.12 | 7.28±2.12 | 7.35±1.94 | 1.28±0.63 | 5.48±1.23 | 1.23±0.48 | 2.01±0.73 | 1.18±0.51 |
| AH28 | 18.44±2.82 | 9.71±2.31 | 10.28±1.83 | 1.71±0.62 | 8.22±2.00 | 1.81±0.42 | 1.82±0.62 | 1.23±0.48 |
| JS137-1 | 1.93±0.46 | 1.01±0.48 | 1.32±0.58 | 1.39±0.61 | 1.37±0.84 | 1.16±0.51 | 1.62±0.74 | 2.44±0.74 |
| KS-1-21 | 7.26±1.72 | 7.71±1.94 | 16.11±3.61 | 2.04±0.54 | 7.07±2.02 | 1.37±0.51 | 1.64±0.72 | 1.99±0.80 |
| LN18 | 16.43±2.14 | 18.92±1.70 | 19.1±2.33 | 17.11±5.62 | 18.65±2.15 | 18.37±2.20 | 5.09±0.98 | 1.56±0.53 |
| LN2 | 15.93±2.17 | 17.36±1.92 | 18.25±2.18 | 11.58±3.34 | 19.64±1.69 | 18.94±2.32 | 4.86±0.97 | 2.33±1.10 |
| LN4 | 12.06±1.87 | 8.55±4.57 | 9.58±1.57 | 12.64±3.63 | 11.39±2.56 | 10.76±3.06 | 4.25±1.05 | 1.78±0.84 |
| LYG50 | 16.67±2.44 | 17.28±1.21 | 13.16±1.69 | 1.96±0.63 | 17.53±2.42 | 1.38±0.83 | 2.20±0.60 | 1.83±0.82 |
| PXO61 | 8.15±2.36 | 12.39±1.64 | 7.43±2.03 | 17.58±5.60 | 9.00±1.82 | 7.94±2.11 | 2.69±0.58 | 2.01±0.45 |
| OS86 | 12.1±1.78 | 10.14±3.80 | 12.51±2.39 | 1.27±0.71 | 10.82±1.86 | 1.89±0.76 | 1.97±0.74 | 1.43±0.76 |
| PXO86 | 16.73±1.69 | 1.15±0.41 | 10.44±2.69 | 10.33±3.61 | 1.65±0.72 | 1.24±0.49 | 9.47±1.84 | 1.96±0.63 |
| PXO99 | 20.62±2.21 | 18.18±1.78 | 11.43±3.10 | 1.60±1.07 | 11.51±2.07 | 1.88±0.95 | 2.17±1.11 | 2.45±0.92 |
| YC12 | 19.04±2.01 | 7.98±3.43 | 17.22±3.30 | 9.52±3.38 | 19.00±1.30 | 11.77±1.24 | 9.06±2.63 | 2.00±0.77 |
| 7914 | 15.84±2.10 | 18.18±2.24 | 17.55±3.22 | 2.18±0.82 | 18.67±1.75 | 1.88±0.90 | 1.86±0.75 | 2.20±1.00 |
| GD1052 | 13.64±1.29 | 15.09±2.13 | 17.37±2.55 | 1.72±0.65 | 8.67±2.45 | 1.3±0.47 | 1.74±0.76 | 1.41±0.64 |
| GD267 | 17.57±1.26 | 16.16±1.77 | 17.53±3.25 | 9.58±3.82 | 11.18±2.46 | 11.28±1.94 | 1.31±0.45 | 2.12±0.84 |
| GD9186 | 10.11±1.56 | 15.14±1.80 | 11.36±1.20 | 2.09±1.05 | 9.57±1.67 | 1.28±0.58 | 1.33±0.62 | 1.96±0.63 |
| GWRXoo3 | 17.89±2.14 | 18.66±1.94 | 17.58±2.71 | 1.94±0.68 | 18.17±2.22 | 1.5±0.44 | 1.36±0.61 | 1.65±0.76 |
| GX4 | 18.74±2.18 | 9.78±3.39 | 16.68±2.64 | 0.95±0.82 | 19.03±2.07 | 2.08±0.54 | 1.78±0.70 | 2.17±0.78 |
| GX49 | 18.98±2.85 | 8.83±3.01 | 17.48±3.06 | 1.50±0.55 | 8.43±2.03 | 1.34±0.50 | 1.83±1.07 | 1.77±0.72 |
| HB03-01 | 19.25±2.26 | 17.31±1.66 | 12.62±1.40 | 2.50±0.85 | 9.74±2.52 | 1.69±0.78 | 2.31±0.76 | 1.23±0.44 |
| HB87-7 | 7.54±2.51 | 4.86±1.29 | 8.62±1.53 | 1.21±0.71 | 8.16±1.80 | 1.02±0.60 | 1.67±0.50 | 2.01±0.75 |
| HNNX3 | 18.9±2.31 | 18.51±2.26 | 17.04±2.76 | 1.96±0.87 | 9.38±2.26 | 1.08±0.44 | 1.94±1.01 | 2.11±0.63 |
| HNZT3 | 18.6±1.30 | 19.14±2.46 | 16.71±2.71 | 1.57±0.68 | 18.31±2.19 | 1.3±0.45 | 1.70±0.68 | 2.02±0.80 |
| HuN37 | 17.78±2.27 | 10.72±4.11 | 17.11±3.00 | 1.68±0.88 | 10.72±2.49 | 1.4±0.50 | 1.29±0.77 | 1.64±0.64 |
| IXO191 | 15.7±1.92 | 18.25±2.32 | 17.16±2.93 | 1.13±0.77 | 9.91±2.07 | 1.32±0.66 | 2.80±0.97 | 1.52±0.62 |
| IXO221 | 11.95±1.85 | 11.72±2.38 | 12.36±1.24 | 16.55±5.37 | 9.11±2.91 | 1.83±1.00 | 7.27±1.35 | 1.39±0.45 |
| JL1 | 19.2±3.67 | 8.15±4.94 | 16.93±2.98 | 15.88±5.01 | 18.39±1.84 | 10.59±1.90 | 2.36±0.71 | 2.24±0.83 |
| JL4 | 18.36±2.00 | 17.15±1.84 | 17.44±3.24 | 17.43±5.51 | 8.65±2.17 | 18.06±2.24 | 2.07±0.73 | 1.29±0.51 |
| JX21 | 2.20±0.72 | 1.55±0.69 | 2.15±3.17 | 2.04±0.42 | 1.32±0.94 | 1.16±0.46 | 1.08±0.45 | 2.04±0.56 |
| 6503 | 1.78±0.53 | 2.00±0.49 | 1.82±3.27 | 1.22±0.81 | 1.51±1.07 | 1.02±0.55 | 1.14±0.56 | 1.10±0.35 |
| LYG48 | 6.86±1.47 | 7.79±3.81 | 9.52±1.66 | 1.82±0.60 | 18.71±1.95 | 1.41±0.82 | 2.06±0.69 | 1.72±0.96 |
| OS26 | 18.58±1.83 | 18.32±2.21 | 12.37±1.11 | 1.66±0.50 | 8.77±1.85 | 2.02±0.67 | 1.95±0.69 | 1.61±0.72 |
| SC-4 | 17.36±2.15 | 18.72±1.92 | 11.58±0.85 | 1.80±0.96 | 18.58±2.46 | 1.79±0.97 | 1.84±0.77 | 0.89±0.40 |
| XZ42 | 17.89±2.03 | 18.63±2.10 | 17.46±3.02 | 2.06±0.68 | 18.52±2.51 | 1.58±0.49 | 2.06±0.97 | 1.81±0.88 |
| XZ44 | 18.21±1.75 | 18.61±2.01 | 17.19±2.74 | 1.80±0.73 | 18.00±2.16 | 1.05±0.47 | 1.82±0.65 | 1.33±0.48 |
| YC1 | 19.32±1.71 | 19.49±2.59 | 13.19±1.35 | 2.20±0.91 | 19.27±2.39 | 2.16±1.21 | 2.23±0.96 | 2.27±1.50 |
| YNYM-5 | 7.40±1.70 | 9.65±3.27 | 12.03±1.50 | 1.21±0.73 | 12.42±2.19 | 1.19±0.54 | 1.28±0.46 | 1.62±0.52 |
| Zhe173 | 2.31±0.85 | 1.56±0.80 | 1.94±3.21 | 1.05±0.63 | 2.07±0.68 | 1.30±0.42 | 1.49±0.58 | 1.82±0.74 |
| JL15 | 17.49±1.91 | 19.07±2.36 | 16.12±2.03 | 16.07±5.32 | 18.17±2.20 | 11.33±2.07 | 2.04±0.71 | 1.90±0.68 |
| DB5 | 18.47±1.88 | 10.1±2.31 | 12.11±1.25 | 10.2±3.63 | 18.98±1.20 | 20.16±1.31 | 2.03±0.73 | 1.30±0.28 |
| DB11 | 15.13±1.65 | 19.13±2.32 | 18.35±2.98 | 15.4±4.83 | 8.47±2.16 | 10.08±2.43 | 1.98±0.69 | 1.45±0.49 |
| DB16 | 19.14±3.07 | 18.9±1.82 | 17.39±2.65 | 17.17±5.45 | 18.57±2.38 | 19.39±2.33 | 1.90±0.67 | 1.76±0.75 |
| DB23 | 19.12±1.84 | 18.32±2.40 | 18.21±3.27 | 16.42±5.12 | 19.03±1.68 | 8.98±2.45 | 1.77±0.81 | 1.74±0.91 |
| TH8 | 16.72±1.7 | 19.72±2.54 | 12.75±1.35 | 17.95±5.78 | 18.66±2.54 | 17.98±2.26 | 1.89±0.91 | 1.11±0.60 |
| TH18 | 18.54±1.8 | 18.91±1.82 | 18.65±3.20 | 16.23±5.14 | 17.29±1.49 | 18.95±2.44 | 1.17±0.56 | 1.73±0.80 |
| DD | 15.4±1.61 | 18.27±1.96 | 17.85±2.93 | 16.92±5.31 | 17.49±2.42 | 19.19±2.15 | 1.14±0.52 | 1.61±1.03 |
| YN04-1 | 11.84±1.51 | 9.50±1.92 | 12.33±1.60 | 2.03±0.86 | 17.49±2.06 | 1.30±0.54 | 1.27±0.61 | 1.78±0.89 |
| JXOV | 11.73±1.38 | 9.36±4.40 | 12.28±1.86 | 1.41±0.63 | 8.76±1.32 | 1.55±0.42 | 1.32±0.55 | 1.56±0.87 |

**Supplemental Table 4 Wild-type rice lines containing different *OsSWEET13* EBEs.**

| **Rice lines** | **Accession ID** | **Origin** | **EBE region** |
| --- | --- | --- | --- |
| IR24 | B232 | China | ATAAAAGCACCACAACTCCCTTC |
| ZS97 | CX241 | - | ATAA--GCACCACAACTCCCTTC |
| Minghui63 | CX145 | China | ATAAA-GCACCACAACACCCTTC |
| American Huangkedao | B018 | USA | TTAAA-GCACCACAACTCCCTTC |
| Xiangdao | B244 | China | ATATA-GCACCACAACTCCCTTC |

**Supplemental Table 5 Lesion lengths (cm) induced by *Xoo* JS137-1, Zhe173, JX21 and 6503 on five near-isogenic rice lines containing *OsSWEET13*.**

| Strains  Rice | JS137-1 | Zhe173 | JX21 | 6503 |
| --- | --- | --- | --- | --- |
| IR24 | 1.60±0.45 | 1.86±0.31 | 1.84±0.49 | 1.70±0.38 |
| ZS97 | 2.11±0.63 | 2.60±0.67 | 1.56±0.89 | 0.95±0.26 |
| Minghui63 | 2.31±0.51 | 2.68±0.62 | 2.69±0.59 | 2.23±0.59 |
| Xiangdao | 5.96±1.90 | 6.72±1.66 | 5.97±1.61 | 1.84±0.76 |
| American Huangkedao | 1.99±0.52 | 2.32±0.64 | 2.74±0.41 | 1.73±0.22 |

**Supplemental Table 6 Features of TALEs and RVDs used in this study.**

| **TALEs** | **Strain** | **Genome Accession** | **Genome positions** | **RVD** |
| --- | --- | --- | --- | --- |
| PthXo1 | PXO99A | NC_010717.2 | 1645241-1649363:-1 | NN-HD-NI-HG-HD-NG-N*-HD-HD-NI-NG-NG-NI-HD-NG-NN-NG-NI-NI-NI-NI-N*-NS-N* |
| PthXo1^*^ | IXO221 | NZ_CP059591.1 | 1704205-1708327:-1 | NN-HD-NI-NG-HD-NG-N*-HD-HD-NI-NG-NG-NI-HD-NG-NN-NG-NI-NI-NI-NI-N*-NS-N* |
| AvrXa23A | LN18 | CP045238.1 | 3969874-3971689:-1 | NI-NN-N*-NG-NS-NN-NN-NN-NI-NN-NI-NG-HD-HD-NI-NG |
|  | LN4 | CP045452.1 | 2735719-2736979:1 | NI-NN-N*-NG-NS-NN-NN-NN-NI-NN-NI-NG-HD-HD-NI-NG |
| PthXo2 | AH28 | NZ_CP074076.1 | 1456789-1460722:-1 | NI-HG-NI-NN-NN-NI-NN-HD-NI-HD-NS-NS-NS-HD-NN-HD-NG-HD-HD-HD-NG-NG |
|  | HuN37 | NZ_CP031456.1 | 4500604-4504534:1 | NI-HG-NI-NN-NN-NI-NN-HD-NI-HD-NS-NS-NS-HD-NN-HD-NG-HD-HD-HD-NG-NG |
| PthXo2B^*^ | JS137-1 | / | / | NI-HG-NI-NN-NN-NI-NN-HD-NI-HD-HD-NS-NS-HD-NN-HD-NG-HD-HD-HD-NG-NG |
| PthXo2B | PXO61 | NZ_CP021789.1 | 4578513-4582308:1 | NI-HG-NI-NN-NI-NN-HD-NI-HD-HD-NS-NS-HD-NI-NI-HD-NG-HD-HD-HD-NG-NG |
| PthXo2C | LN18 | CP045238.1 | 2288799-2288868:1 | NI-HG-NI-NN-NI-NN-HD-HD-HD-HD-NS-NS-HD-NI-NI-HD-NG-HD-HD-HD-NG-NG |
|  | LN4 | CP045452.1 | 739718-740477:-1 | NI-HG-NI-NN-NI-NN-HD-HD-HD-HD-NS-NS-HD-NI-NI-HD-NG-HD-HD-HD-NG-NG |
| PthXo3 | PXO61 | NZ_CP021789.1 | 3258770-3263276:1 | NI-HG-NI-HG-NI-NI-NI-HD-NN-HD-HD-HD-NG-HD-n*-NI-HD-HD-NN-NS-NI-NN-NN-NG-NN-HD-N*-NS-N* |
| PthXo3 | AH28 | NZ_CP074076.1 | 2749136-2753057:-1 | NI-HG-NI-HG-NI-NI-NI-HD-NN-HD-HD-HD-NG-HD-n*-NI-HD-HD-NN-NS-NI-NN-NN-NG-NN-HD-N*-NS-N* |
| PthXo3* | IXO221 | NZ_CP059591.1 | 2608550-2612264:1 | NI-HG-NI-HG-NI-NI-NI-HD-NN-HD-NS-NG-SS-HD-NI-NI-NN-NI-NN-NI-NG |
| PthXo3* | HuN37 | NZ_CP031456.1 | 3179401-3183847:1 | NI-HG-NI-NI-NS-HD-NN-HD-HD-HD-NS-HD-N*-NI-HD-HD-NN-NS-NN-NN-NG-NN-HD-N*-NS-NS-N* |
| AvrXa7 | LN18 | CP045238.1 | 976318-978622:1 | NI-HG-NI-NI-NS-HD-NN-HD-HD-HD-NS-N*-N*-HD-HD-NS-NS-NN-NN-NI-NG-NN-NI-N*-NS-N* |
|  | LN4 | CP045452.1 | 739718-740477:-1 | NI-HG-NI-NI-NS-HD-NN-HD-HD-HD-NS-N*-N*-HD-HD-NS-NS-NN-NN-NI-NG-NN-NI-N*-NS-N* |
|  | PXO86 | NZ_CP007166.1 | 1800158-1804499:-1 | NI-HG-NI-NI-NS-HD-NN-HD-HD-HD-NS-N*-N*-HD-HD-NS-NS-NN-NN-NI-NG-NN-NI-N*-NS-N* |

**Supplemental Table 7 Bacterial strains and plasmids used in this study**

| **Plasmid or Strain** | **Antibiotic** | **Description** | **Source** |
| --- | --- | --- | --- |
| Plasmids |  |  |  |
| pBy02-OsCas9 | Sm+Km | CRISPR Cas9 system Cas9 vector | (Xu et al., 2019) |
| pENTR4-gRNA4 | Sm+Km | CRISPR Cas9 system guideRNA (gRNA) vector | (Xu et al., 2019) |
| pENTR4-gRNA5 | Sm+Km | CRISPR Cas9 system gRNA5 vector | (Xu et al., 2019) |
| pgRNA4-SW14 | Km | pENTR4-gRNA4 containing gRNA SW14 | This study |
| pgRNA4-SW11 | Km | pENTR4-gRNA4 containing gRNA SW11 | This study |
| pgRNA4-SW13 | Km | pENTR4-gRNA4 containing gRNA SW13 | This study |
| pgRNA4-SW1314 | Km | pENTR4-gRNA4 containing gRNA SW13 and SW14 | This study |
| pgRNA4-SW1113 | Km | pENTR4-gRNA4 containing gRNA SW11 and SW13 | This study |
| pgRNA4-SW1114 | Km | pENTR4-gRNA4 containing gRNA SW11 and SW13 | This study |
| pgRNA5-SW111413 | Km | pgRNA4-SW1114 and pgRNA5-SW13 as a *Hin*dIII fragment | This study |
| pCg4-SW11 | Km | pBY02-OsCas9 containing gRNA SW11 | This study |
| pCg4-SW13 | Km | pBY02-OsCas9 containing gRNA SW13 | This study |
| pCg4-SW14 | Km | pBY02-OsCas9 containing gRNA SW14 | This study |
| pCg4-SW1113 | Km | pBY02-OsCas9 containing gRNA SW11 and SW13 | This study |
| pCg4-SW1314 | Km | pBY02-OsCas9 containing gRNA SW13 and SW14 | This study |
| pCg4-SW1114 | Km | pBY02-OsCas9 with gRNA SW11, SW14 | This study |
| pCg5-SW111413 | Km | pBY02-OsCas9 with gRNA SW11, SW14 and SW13 | This study |
|  |  |  |  |
| Bacterial strains |  |  |  |
| *E. coli* DH5α | Null | F-*endA1*, *thi-1*, *recA1*, Φ80*lacZ*, ΔM15 | TransGen Biotech |
| *A. tumefaciens* |  |  |  |
| EHA105 | Rif | pTic58DT-DNA | ToloBio |
| EHA105[pCg4-SW11] | Km+Rif | EHA105 containing pCg4-SW11 | This study |
| EHA105[pCg4-SW14] | Km+Rif | EHA105 containing pCg4-SW14 | This study |
| EHA105[pCg4-SW14] | Km+Rif | EHA105 containing pCg4-SW13 | This study |
| EHA105[pCg4-SW1314] | Km+Rif | EHA105 containing pCg4-SW1314 | This study |
| EHA105[pCg4-SW1113] | Km+Rif | EHA105 containing pCg4-SW1113 | This study |
| EHA105[pCg4-SW111413] | Km+Rif | EHA105 containing pCg4-SW111413 | This study |

Abbreviations: Km, kanamycin; Rif, rifampicin; Sm, streptomycin.

**Supplemental Table 8. Guide RNA design based on three *OsSWEET* promoter regions.**

| **Gene** | **First 400 bp of promoter sequence^1^** | **Oligo DNA sequence^2^** |
| --- | --- | --- |
| ***OsSWEET11a*** | ATGACCACATATTCAGAGTAGTGGAGAGAGGGACAGATCTAGAGGTAGAAAAAGAAAATTCATATAAATGATATATCAGAGTGAAAAAGAAATATCAAGCACAAGAAAAAAAAGCAAAGGTTAGATATGCATCTCC**CCC**TACTGTACACCACCAAAAGTGGAGGGTCTCCAACTATATAAACACTGAGCCATGGCCAAGGCCAAACCACACATGCAGTTGTAGTAGCACTTAAGCCTTCCTCTCTAGCTAGCATCTCTTGTGTCAGGAAGTTGGAAGGGATTTCTGGCTAGTTTCTAGCTGGTGTCTCCTCTCCTCTTCCTAACCTTCTCACTGATTAACACCTTAGAGTTAGTTAATAACCTTCATCACCAGTAGCA | SW11-F: TGTTGACTTTTGGTGGTGTACAGTA  SW11-R: AAACTACTGTACACCACCAAAAGTC |
| ***OsSWEET13*** | CAGTAGCTCATTTTTGTAAAAGCCTAATTATTGTGCGTGTCCAAAAGACTTTCCTCAAAAGCAAATAAAGAAAAAAAATCTTTGCATAATTATTCTATGATTACTTTGATGCGTACGTGAATGGCCATGGGTAGGAGGCAACCAAGTGATTCCCACCTAGCTAGCTTTGCT**CCT**ATATAAAGCACCACAACTCCCTTCATTCCTCTCCAAGAGTTTTCAGCCAACACATTGAACTCTTCTTCAGAGCTCTCCCTTCCCTCCACAAAGGGGGTCTAGGGTTAGAGTGTGTGTGTCTGTGACAAGTTCCAAGCTAGCAACAACAAGCTCAATTCCTTGCTTGTTTGCTTCCATATTACACTACATCTCTTCCCTTCAATTACCCCCCTTTTAGCACACAAAA | SW13-F1:  TGTTGGAGTTGTGGTGCTTTATAT  SW13-R1:  AAACATATAAAGCACCACAACTCC  SW13-F2:  GTGTGGAGTTGTGGTGCTTTATAT  SW13-R2:  AAACATATAAAGCACCACAACTCC |
| ***OsSWEET14*** | AGTTTGTGTGTGCAGCTATATTGCCTATTGGTGTCCAGGGTCACACACCATAAGGGCATGCATGTCAGCAGCTGGTCATGTGTGCCTTTTCATTCCCTTCTTCCTTCCTAGCACTATATAAA**CCC**CCTCCAACCAGGTGCTAAGCTCATCAAGCCTTCAAGCAAAGCAAACTCAAGTAGTAGCTGATTACCAGCTCTTCTCTCTTCTCATTGAGAAGAGGGAATTAAGTTTTGATCTCTGCTTTATTGCCTGATCATCCTCTTGTTACTTGCAAGCAAGAACAGTAGTGTACTGTGCCTCATTGATCTCCTCCCACCAAACTCTCTCTCTCTCTCTCATATTCCGAGCTAGCTAGTTAATCAAGATCTTGCTGCA | SW14-F:  GTGTGCTTAGCACCTGGTTGGAGG  SW14-R:  AAACCCTCCAACCAGGTGCTAAGC |

^1^The EBEs recognized by PthXo1, PthXo2 and PthXo3 are highlighted in yellow, and the EBE recognized by AvrXa7 is underlined. The sequences targeted for mutation and the protospacer adjacent motif (PAM) of Cas9/gRNA are shown in red.

^2^ The oligonucleotide sequences in blue font were paired with *Btg*ZI-digested vector, and sequences colored in green were paired with *Bsa*I-digested vector.

**Supplemental Table 9 Primers used in this study**

| Primer | 5' to 3' Sequence | Purpose or Function |
| --- | --- | --- |
| U6P-F | AAGAACGAACTAAGCCGGAC | gRNA4 universal primer |
| gRNA4-F | CCTACCGAACAATGAAGAACCC | gRNA4 universal primer |
| gRNA4-R | ACACAACCACCAAATACAGCCA | gRNA4 universal primer |
| Cas9-F | GGGTAATGAACTCGCTCTGC | CRISPR Cas9 universal primer |
| Cas9-R | TGGCGTCAAGAACTTCCTTTG | CRISPR Cas9 universal primer |
| SW11-F1 | TGTTGACTTTTGGTGGTGTACAGTA | Construction and identification of SW11(*Btg*ZI) mutants |
| SW11-R1 | AAACTACTGTACACCACCAAAAGTC | Construction and identification of SW11(*Btg*ZI) mutants |
| SW14-F2 | GTGTGCTTAGCACCTGGTTGGAGG | Construction and identification of SW14(*Bsa*I) mutants |
| SW14-R2 | AAACCCTCCAACCAGGTGCTAAGC | Construction and identification of SW14(*Bsa*I) mutants |
| SW13-F1 | TGTTGGAGTTGTGGTGCTTTATAT | Construction and identification of SW13(*Btg*ZI) mutants |
| SW13-R1/2 | AAACATATAAAGCACCACAACTCC | Construction and identification of SW13(*Btg*ZI/*Bsa*I) mutants |
| SW13-F2 | GTGTGGAGTTGTGGTGCTTTATAT | Construction and identification of SW13(*Bsa*I) mutants |
| SW11p-F | GGTGTTAATCAGTGAGAAGG | Detecting nucleotide changes in *OsSWEET11a* promoter |
| SW11p-R | GAGAGGGACAGATCTAGAGGTAG | Detecting nucleotide changes in *OsSWEET11a* promoter |
| SW13p-F | ATGGCTAGTGAGAGGTGC | Detecting nucleotide changes in *OsSWEET13* promoter |
| SW13p-R | AGGAATTGAGCTTGTTGTT | Detecting nucleotide changes in *OsSWEET13* promoter |
| SW14p-F | AGCTTGCCCAACTCTAGATC | Detecting nucleotide changes in *OsSWEET14* promoter |
| SW14p-R | ACACTACTGTTCTTGCTTGC | Detecting nucleotide changes in O*sSWEET14* promoter |
| OsActin qRT-F | GTTCCTGCTGTTTGTTCTGTTG | Amplifying fragment of *OsActin* |
| OsActin qRT-R | ATCTCACGCATTACCCTACCTT | Amplifying fragment of *OsActin* |
| OsSWEET11a-qRT-F | AGTCGACGGGAGGGTACAG | Amplifying fragment of *OsSWEET11* |
| OsSWEET11a-qRT-R | TGATGGTCAGCAGCGGC | Amplifying fragment of *OsSWEET11* |
| OsSWEET13-qRT-F | TCCTTGGCAACCTCATATCC | Amplifying fragment of *OsSWEET13* |
| OsSWEET13-qRT-R | GATCAGCGCGTAGAAGATCC | Amplifying fragment of *OsSWEET13* |
| OsSWEET14-qRT-F | TCTACGCCCCCAAGAAGGCCA | Amplifying fragment of *OsSWEET14* |
| OsSWEET14-qRT-R | ACCCAACCAAGAACCACGATGC | Amplifying fragment of *OsSWEET14* |

**References**

HOPKINS, C. M., WHITE, F. F., CHOI, S. H., GUO, A. & LEACH, J. E. 1992. Identification of a family of avirulence genes from Xanthomonas oryzae pv. oryzae. *Mol Plant Microbe Interact,* 5**,** 451-9.

JI, Z., JI, C., LIU, B., ZOU, L., CHEN, G. & YANG, B. 2016. Interfering TAL effectors of Xanthomonas oryzae neutralize R-gene-mediated plant disease resistance. *Nature Communications,* 7**,** 13435-13435.

XU, Z., WANG, S., LIU, L., YANG, Y., ZHU, B., ZOU, L. & CHEN, G. 2020. Genome Resource of a Hypervirulent Strain LN4 of Xanthomonas oryzae pv. oryzae Causing Bacterial Blight of Rice. *Plant Disease,* 104**,** 2764-2767.

XU, Z., XU, X., GONG, Q., LI, Z., LI, Y., WANG, S., YANG, Y., MA, W., LIU, L., ZHU, B., ZOU, L. & CHEN, G. 2019. Engineering Broad-Spectrum Bacterial Blight Resistance by Simultaneously Disrupting Variable TALE-Binding Elements of Multiple Susceptibility Genes in Rice. *Molecular Plant,* 12**,** 1434-1446.

XU, Z., XU, X., WANG, Y., LIU, L., LI, Y., YANG, Y., LIU, L., ZOU, L. & CHEN, G. 2022. A varied AvrXa23-like TALE enables the bacterial blight pathogen to avoid being trapped by Xa23 resistance gene in rice. *Journal of Advanced Research*.

YANG, B. & WHITE, F. F. 2004. Diverse members of the AvrBs3/PthA family of type III effectors are major virulence determinants in bacterial blight disease of rice. *Mol Plant Microbe Interact,* 17**,** 1192-200.
